# A dual CEST-fluorescent reporter enables cross-validated tracking of hematopoietic engraftment in deep bone marrow

**DOI:** 10.7150/thno.140697

**Published:** 2026-09-24

**Authors:** Tehila Alter, Narmeen Haj, Doaa Ali-Naffaa, Maya Rom, Ramez Daniel, Galit Saar, Assaf A. Gilad, Katrien Vandoorne

**Affiliations:** 1 Faculty of Biomedical Engineering, Technion-Israel Institute of Technology, Haifa, Israel.; 2 Biomedical Core Facility, Rappaport Faculty of Medicine, Technion-Israel Institute of Technology, Haifa, Israel.; 3 Biomedical Engineering Faculty, Tel Aviv University, Tel Aviv, Israel.; 4 Chemical Engineering and Radiology, Michigan State University, East Lansing, USA.

**Keywords:** CEST-MRI, reporter gene imaging, hematopoietic stem and progenitor cells, bone marrow engraftment, cell tracking

## Abstract

Reporter gene imaging can enable detection of engineered cells *in vivo*, but tracking hematopoietic engraftment within deep bone marrow (BM) remains challenging. We addressed this challenge by developing a dual CEST-MRI and fluorescent reporter system for cross-validated detection of reporter-positive donor-derived hematopoietic cells and their progeny in BM, with fluorescence-based assessment of HSPC-enriched compartments.

**Methods:**

A lentiviral dual-reporter cassette co-expressing the red fluorescent protein mScarlet-I (mScarlet) and the CEST-MRI reporter SuperCESTide (SC) via a T2A linker was evaluated in CT26 colon carcinoma cells *in vitro* and in a bilateral flank tumor model with contralateral reporter-transduced and non-transduced tumors in BALB/c mice (n = 6 tumors per group). *In vivo* optical imaging, CEST-MRI, *ex vivo* optical imaging, fluorescence microscopy, and histology were used for validation. The platform was then applied to primary murine hematopoietic lineage-negative (Lin⁻) cells and Lin⁻Sca-1⁺c-Kit⁺ (LSKs), with reporter expression analyzed by fluorescence microscopy and flow cytometry. Reporter-transduced Lin⁻ cells were transplanted into C57BL/6J mice after half-body irradiation, and engraftment was assessed 8 weeks later by whole-body fluorescence imaging, femoral CEST-MRI, intravital calvarial microscopy, *ex vivo* confocal microscopy, optical imaging, and flow cytometry. Femoral CEST-MRI was compared among non-irradiated controls, irradiated recipients transplanted with non-reporter-transduced Lin⁻ cells, and irradiated recipients transplanted with mScarlet-SC reporter-transduced Lin⁻ cells.

**Results:**

The dual mScarlet-SuperCESTide reporter showed efficient expression in CT26 cells and generated detectable red fluorescence and increased CEST-MRI contrast in reporter-positive tumors compared with contralateral non-transduced controls. *Ex vivo* optical imaging and histology confirmed reporter expression in tumor tissue. In murine BM-derived Lin⁻ cells, reporter expression was detected by fluorescence microscopy and flow cytometry, supporting feasibility in sensitive hematopoietic populations. Following transplantation, reporter-transduced Lin⁻ cells were detected eight weeks after engraftment using fluorescence imaging and femoral CEST-MRI. The recipients with reporter-transduced Lin⁻ cells showed increased femoral CEST contrast associated with the reporter compared with both irradiated recipients transplanted with non-transduced Lin⁻ cells and non-irradiated BM controls. Additionally, intravital microscopy visualized mScarlet⁺ cells within the BM niches of the calvarium. *Ex vivo* confocal microscopy and optical imaging confirmed reporter retention in BM, and flow cytometry confirmed mScarlet signal within Lin⁻ and LSK hematopoietic compartments in reporter-transduced recipients compared with non-transduced controls.

**Conclusion:**

We developed a dual CEST-fluorescent reporter platform that enables cross-validated imaging of reporter-transduced hematopoietic cells in deep BM. By integrating noninvasive CEST-MRI with fluorescence-based cellular validation, this feasibility study establishes a preclinical framework for cross-validated, noninvasive assessment of engineered hematopoietic cell localization and persistence *in vivo* and supports future evaluation of the platform for serial imaging.

## Introduction

Hematopoietic stem and progenitor cells (HSPCs) are self-renewing cells that sustain blood and immune cell production throughout life. Because they persist over the long term and continuously seed downstream progeny, genetic or epigenetic lesions acquired in HSPCs can propagate throughout the hematopoietic system and contribute to serious disease 1. This central role has made HSPCs a major focus of therapeutic engineering: *ex vivo* gene addition and gene editing of autologous HSPCs is reshaping treatment for monogenic hematologic and immunologic disorders, and next-generation approaches increasingly aim to enhance or enable HSPC modification *in vivo* using viral delivery platforms 1-4. Yet, in both preclinical studies and clinical practice, donor cell persistence is most often inferred indirectly from peripheral blood recovery/chimerism and periodic invasive marrow sampling rather than measured by longitudinal, whole-skeleton imaging within the deep bone marrow (BM) 5,6. This limits mechanistic studies of niche-HSPC interactions and complicates the development of engineered hematopoietic therapies, in which investigators need to verify that viable, genetically defined donor cells persist in the marrow over time.

In mice, intravital microscopy enables high-resolution visualization of calvarial marrow niches and inflammatory cell dynamics, but it is semi-invasive, restricted to the calvaria, and not well suited for quantitative assessment across the skeleton’s marrow 7-10. Optical reporters remain indispensable for confirming genetic manipulation and enabling cell-level validation *ex vivo*, but light scattering and absorption, particularly through mineralized tissue, limit quantitative assessment of deep marrow. MRI reporter gene strategies offer depth penetration and the potential to detect viable cells noninvasively 11,12, but have not become routine for tracking hematopoietic populations within the BM.

Among MRI reporter approaches, chemical exchange saturation transfer (CEST) MRI is attractive because it generates molecular contrast from exchangeable protons without ionizing radiation. Early genetically encoded CEST reporters based on lysine-rich proteins demonstrated *in vivo* feasibility but were limited by repetitive sequence design 13-16. Computational genetic programming was introduced as a framework for engineering MRI reporter genes 13. Fillion *et al*. subsequently used in silico peptide optimization to construct and physically characterize SuperCESTide, a next-generation CEST-MRI reporter with broadened amino-acid composition and reduced lysine content 17. More recently, systemic rAAV-mediated delivery of SuperCESTide demonstrated that reporter-associated CEST contrast can be detected in deep tissue 18. However, CEST measurements remain susceptible to endogenous background signals and technical confounders, including B₀/B₁ inhomogeneity, highlighting the need for rigorous orthogonal validation of reporter-associated contrast 16. To date, SuperCESTide-enabled CEST-MRI has been demonstrated only in hepatocytes, which are large, metabolically active, and abundant within the imaged tissue 18. Detecting reporter-expressing HSPCs in BM is substantially more challenging because these cells are small, more rare, and dispersed throughout a heterogeneous marrow microenvironment containing adipocytes, stromal cells, and diverse endogenous hematopoietic populations. Sensitive reporter systems are therefore needed to overcome endogenous CEST background and reliably detect rare, dispersed HSPCs in BM. Here, we developed a dual CEST–fluorescent reporter comprising both SuperCESTide 17,18 and the red fluorescent protein mScarlet-I (mScarlet), enabling constitutive co-expression of both reporters from a single transcript. We first validated reporter expression in CT26 murine colon carcinoma cells and used a within-animal bilateral flank-tumor model to assess reporter detectability by optical imaging and CEST-MRI. We then applied the platform to primary murine hematopoietic cells^19^,^20^ and evaluated the *in vivo* detectability and marrow persistence of transplanted reporter-transduced donor cells using complementary whole-body optical imaging, femoral CEST-MRI, and calvarial intravital microscopy. Together, these experiments provide a cross-validation framework linking deep-tissue CEST-MRI contrast with verified cellular reporter expression and support the feasibility of noninvasively imaging reporter-transduced hematopoietic cells in BM.

## Materials and Methods

### Cells, vector design and transduction

The following cell lines were used: HEK293T (ATCC CRL-3216) for lentiviral production, CT26 murine colon carcinoma cells (ATCC CRL-2638) and primary BM cells harvested from femurs and tibias of donor C57BL/6J mice (Envigo, 7-9 weeks; see below). Cells were regularly tested for mycoplasma using MycoStrip® (InvivoGen, rep-mys-10). The lentiviral dual-reporter cassette encoded mScarlet-I (hereafter, mScarlet) linked via a T2A peptide to the CEST-MRI reporter SuperCESTide (SC) 17; the combined cassette is referred to as mScarlet-SC (**Figure 1A**). Cells were analyzed in three groups: (i) mScarlet-SC dual-reporter-transduced, (ii) tdTomato-only transduced, and (iii) non-transduced controls. The tdTomato construct was used only as an *in vitro* fluorescent transduction control and was not included in the *in vivo* IVIS studies. Because tdTomato and mScarlet differ in brightness and spectral properties, tdTomato was not used for quantitative fluorescence comparisons. Non-transduced cells served as the primary negative control for reporter-associated fluorescence and CEST-MRI contrast.

HEK293T cells were maintained in high-glucose Dulbecco’s modified Eagle medium (DMEM; 4.5 g/L D-glucose; Sigma-Aldrich, D6429) supplemented with 10% fetal bovine serum (FBS) and 1% penicillin/streptomycin. For lentiviral production, HEK293T cells at 70%-90% confluence were transfected with CalFectin (SignaGen, SL100478) using the expression plasmid (5 µg), ΔNRF, and VSV-G (2.8 µg each). Viral supernatants were collected at 48 and 72 h, clarified, and filtered through a 0.45 µm filter. Viral input was adjusted empirically to achieve 60-80% transduction efficiency.

CT26 cells were cultured in RPMI-1640 (Sigma-Aldrich) supplemented with 10% FBS and 1% penicillin/streptomycin. Cells were seeded at 1.5-2 × 10^5^ cells per well in 6-well plates and transduced 24 h later with viral supernatant diluted in serum- and antibiotic-free medium. After 18-24 h incubation with lentivirus, cells were washed three times with PBS and returned to fresh complete medium. Reporter expression was assessed 48-72 h after transduction by brightfield and fluorescence microscopy.

For primary hematopoietic studies, murine BM was harvested from C57BL/6J mice (see below) into PBS containing 0.5% bovine serum albumin using centrifugation-based extraction. Single-cell suspensions were filtered through a 70 µm nylon mesh strainer and subjected to red blood cell lysis using 1× RBC lysis buffer (BioLegend; 2 mL per bone). Lysis was neutralized with 10 mL PBS per bone. Lineage-negative (Lin⁻) cells were enriched using the Lineage Cell Depletion Kit (Miltenyi Biotec, 130-090-858) in MACS buffer consisting of PBS, 0.5% BSA, and 2 mM EDTA (Sigma, 324506). Cells were incubated with a biotinylated lineage antibody cocktail, labeled with anti-biotin microbeads, and separated on MS/LS columns using MiniMACS, MidiMACS, or OctoMACS separators (Miltenyi Biotec, 130-042-501 and 130-042-108). The Lin⁻ -flow-through was collected, whereas Lin^+^ cells were retained in the magnetic field. Lin⁻ cells were transduced in viral supernatant for 20-23 h, washed, and allowed to recover in serum-free, antibiotic-free StemSpan SFEM medium (STEMCELL Technologies, 09600) prior to downstream analyses. To evaluate initial transduction efficiency and reporter expression prior to transplantation, freshly isolated lineage-negative (Lin⁻) and Lin⁻Sca-1⁺c-Kit⁺ (LSK) hematopoietic cells were analyzed approximately 24 h after lentiviral transduction.

### Cell culture microscopy

Reporter expression was assessed by fluorescence microscopy using an EVOS M5000 imaging system (Invitrogen, AMF5000). Images were analyzed in Fiji (ImageJ) by applying the default fluorescence threshold and measuring threshold-positive area. For quantification, 250 × 250 µm ROIs were analyzed across five independent experiments. The fluorescent area was normalized to the total field area and reported as the percent fluorescence-positive area per ROI.

### Flow cytometry

For HSPC immunophenotyping, freshly isolated BM cells were incubated with a biotinylated lineage antibody cocktail against CD5, CD45R (B220), CD11b, Gr-1 (Ly-6G/C), 7-4, and Ter-119 (Miltenyi Biotec; 1:300). Cells were then stained with streptavidin-APC (BioLegend, 405207) for Lin^+^, Brilliant Violet 421^TM^ anti-mouse CD117 (c-Kit) (BioLegend, 105828, clone 2B8), and PE/Cyanine7 (PE-Cy7) anti-mouse Ly-6A/E (Sca-1) Antibody (BioLegend, 108114, clone D7), each at 1:300 dilution. Data were acquired on a Cytek Aurora spectral flow cytometer equipped with 405, 488, 561, and 635 nm lasers. Lin⁻ cells were defined as lineage-negative events, and LSK cells were identified as Lin⁻Sca-1⁺c-Kit⁺. Data were analyzed using FlowJo v10.7.1 (BD Biosciences).

Imaging flow cytometry was performed on an ImageStreamX MkII imaging cytometer to quantify reporter expression at the single-cell level and to obtain representative cell images. Samples included lentivirally transduced Lin⁻ BM cells and, where indicated, antibody-defined LSK cells. Cells were washed, resuspended in PBS or FACS buffer, and acquired using a 40× objective with brightfield and fluorescence channels. Red fluorescence from mScarlet or tdTomato was excited using the 561 nm laser and collected in the appropriate emission channel. Side scatter was acquired using the 785 nm laser. Compensation or spectral unmixing was established using single-color controls as appropriate. Data were analyzed in IDEAS using a standardized gating pipeline consisting of: (i) focus gating based on gradient RMS, (ii) debris exclusion by brightfield area, (iii) singlet selection by aspect ratio versus area, and (iv) quantification of reporter-positive cells based on red fluorescence intensity. Representative single-cell images were exported for figures.

### Mouse models

All animal procedures were approved by the Technion Institutional Animal Care and Use Committee (IACUC; protocols IL-014-01-23 and IL-104-07-25) and were performed in accordance with institutional and national guidelines. Unless otherwise stated, mice were housed under specific-pathogen-free (SPF) conditions with ad libitum access to food and water and were anesthetized with isoflurane (2-3% for induction; 1-2% for maintenance) for all procedures and *in vivo* imaging experiments.

**Bilateral CT26 flank tumor model for *in vivo* reporter expression.** Female BALB/c mice (Envigo, 7-9 weeks) were used for syngeneic bilateral flank tumor implantation. CT26 cells were either lentivirally transduced with the mScarlet-SC construct or left non-transduced. Each mouse received subcutaneous injections of 4 × 10^5^ cells per site in 100 µL sterile PBS, with non-transduced CT26 cells implanted in the left flank and reporter-transduced CT26 cells implanted contralaterally. Injections were performed using a 27G needle. Tumor growth and body weight were monitored every 2-3 days. Tumors were allowed to grow for 3 weeks prior to imaging. At the imaging time point, mice underwent whole-body fluorescence imaging and CEST-MRI.

**Half-body irradiation and transplantation model.** Female C57BL/6J mice (Envigo, 7**-**9 weeks) were used as BM donors and transplantation recipients. Donor mice were euthanized by cervical dislocation for isolation of Lin⁻ BM cells. Recipient mice were anesthetized by intraperitoneal injection of a ketamine/xylazine mixture prepared as 1 mL ketamine, 0.25 mL xylazine, and 8.75 mL sterile saline, administered at 0.1 mL per 10 g body weight. To establish a localized engraftment niche while reducing required donor cell dose and enabling within-animal comparisons, recipients underwent left-sided half-body irradiation (900 cGy) using an X-Rad320 Biological Irradiator (Precision X-Ray Inc., Madison, CT). The contralateral side was shielded with a 3-mm lead barrier 21. Irradiation was performed at a 40 cm source-to-skin distance using filter set #2 (1.5 mm Al, 0.25 mm Cu, and 0.75 mm Sn). Seven hours later, mice received retro-orbital injections of 1-2× 10^6^ lentivirally transduced Lin⁻ cells suspended in 200 µL RPMI using a 31G needle. Experimental groups were (i) non-irradiated controls, (ii) irradiated recipients transplanted with non-transduced Lin⁻ cells, and (iii) irradiated recipients transplanted with mScarlet-SC-transduced Lin⁻ cells. Mice were monitored daily and imaged 8 weeks after transplantation.

### Optical imaging

*In vivo* fluorescence imaging was performed using an IVIS Lumina X5 system (PerkinElmer) in auto-exposure mode. Although mScarlet-I has fixed intrinsic spectral properties, tissue-dependent scattering and autofluorescence can influence the *in vivo* fluorescence signal-to-background ratio and the optimal filter selection 22. Therefore, mScarlet-I fluorescence was acquired using model-specific filter pairs selected during preliminary testing: Ex 580 nm/Em 620 nm for the CT26 bilateral flank-tumor model and Ex 520 nm/Em 620 nm for the Lin⁻ transplantation model. The reported wavelengths represent the center wavelengths of band-pass filters with 20-nm excitation and 40-nm emission bandwidths: Ex 570-590 nm/Em 600-640 nm for the CT26 model and Ex 510-530 nm/Em 600-640 nm for the Lin⁻ transplantation model. Thus, both filter combinations detect mScarlet-I while sampling different portions of its excitation spectrum. The same filter pair and imaging/analysis procedures were used for all animals within each experimental model. Because different filter pairs were used, fluorescence intensities from the CT26 and Lin⁻ models were not compared directly. For CT26 bilateral flank tumors, IVIS imaging was performed 3 weeks post-implantation. Reporter-transduced and non-transduced tumors from the same mouse were imaged in the same field using identical filter and acquisition settings. ROIs were drawn over each tumor, and radiant efficiency was quantified after background normalization to adjacent non-tumor tissue. For Lin⁻ transplantation studies, IVIS imaging was performed 8 weeks post-transplantation. ROIs were placed over marrow-rich regions, including the femur, pelvis, and skull, using Living Image v4.7.2. Signal was reported as background-subtracted radiant efficiency ([photons/s/cm²/sr]/[µW/cm²]). For *ex vivo* optical imaging, excised CT26 tumors and tibias from transplanted mice were rinsed in PBS and imaged using a MILabs Vector7 optical/CT system (Ex 527 nm/Em 615 nm; 6000-ms exposure; f/4.0). Tumor and tibia ROIs were quantified in Fiji after background subtraction. Matched samples were acquired and analyzed using identical settings.

### *In vivo* MRI

CEST-MRI was performed on a 9.4T horizontal bore scanner (Bruker BioSpec, Ettlingen, Germany). Mice were anesthetized with 1.5% isoflurane in oxygen (0.7 L/min) and placed on a heated platform (37°C). Respiration was monitored throughout imaging (Small Animal Instruments, Stony Brook, NY). For imaging of flank tumors, a cylindrical volume coil (86 mm inner diameter) was used for transmission and detection. For the irradiated / healthy femur, mice were positioned in right lateral decubitus with a surface coil (20 mm diameter) placed over the left femur.

The imaging protocol consisted of three sequential acquisitions: **(1)** T2-weighted anatomical imaging using a Rapid Acquisition with Relaxation Enhancement (RARE) sequence with repetition time (TR) = 2500 ms, echo time (TE) = 30 ms, RARE factor = 4, field of view (FOV) = 4 × 4 cm², matrix = 256 × 256, in-plane resolution = 156 µm, 3 slices of 1 mm thickness, 4 averages, and an acquisition time of *≈* 5 min; **(2)** CEST imaging using a RARE-based acquisition with TR = 6000 ms, effective TE = 19.4 ms, RARE factor = 10, FOV = 4 × 4 cm², matrix = 64 × 64, in-plane resolution = 625 µm, slice thickness = 1 mm, saturation power = 2 µT, and a single 4 s Hermite saturation pulse per TR (66.7% temporal duty cycle) applied at frequency offsets ranging from +5.25 to -5.25 ppm in 0.25 ppm steps, with 43 offset acquisitions plus one unsaturated S₀ acquisition and a total scan time of ≈26–27 min; and **(3)** WASSR (water saturation shift referencing) acquisition for B0 correction, performed using parameters similar to the CEST scan, with offsets ranging from +1 to -1 ppm in 0.1 ppm steps, 21 repetitions, and an acquisition time of *≈* 12 min.

All Z-spectral data were processed using custom-written MATLAB scripts, including scripts developed by the research group of Dr. Moritz Zaiss, publicly available on GitHub at https://github.com/cest-sources. In short, to correct for B_0_ inhomogeneity, for each voxel, the WASSR spectrum was used to identify the frequency offset corresponding to the minimum signal (water resonance), and the CEST Z-spectrum was shifted accordingly prior to analysis.

Claude (Anthropic) was used to assist in adapting, refining, and debugging existing code.

### CEST-MRI processing and asymmetrical magnetization transfer ratio analysis

CEST data were acquired over a saturation offset range of -5.25 to +5.25 ppm in 0.25 ppm steps. Z-spectra were generated by normalizing the signal measured at each saturation offset (S(Δω)) to the unsaturated reference image (S0). After voxel-wise B0 correction using WASSR, CEST contrast was expressed using the magnetization transfer ratio asymmetry (MTR_asym_), which provides an asymmetry-based estimate of saturation transfer by comparing signal attenuation at equal and opposite frequency offsets around the water resonance:



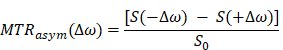



Conceptually, MTR_asym_ highlights downfield CEST effects (e.g., from exchangeable protons) by subtracting the corresponding upfield signal, thereby partially compensating for symmetric contributions such as direct water saturation and broad magnetization transfer.

For tumor, BM or muscle analyses, ROI-averaged MTR_asym_ spectra were extracted from manually defined ROIs. Integrated CEST contrast was quantified as the area under the ROI-averaged MTRasym spectrum between 1 and 2 ppm (%·ppm). This interval was selected as a conservative reporter-weighted range because it captured the dominant downfield group difference while reducing sensitivity to direct water saturation at very low offsets and to broader asymmetric background effects, including semisolid magnetization-transfer asymmetry and NOE-related contributions 23,24. In the femoral marrow data, marked differences were observed below 1 ppm, particularly between irradiated and non-irradiated groups. Because these low-offset differences could reflect direct water saturation, residual B₀ misregistration, and irradiation-related changes in endogenous CEST or magnetization-transfer signal, offsets below 1 ppm were excluded from the primary AUC analysis. This restriction reduces but does not eliminate the endogenous background; the control groups were used to mitigate its effects. For display purposes, representative CEST contrast maps were generated at 1.8 ppm, selected based on the observed trend toward increased reporter-associated CEST contrast at this offset and its position within the 1-2 ppm window used for quantitative analysis.

### Tumor histology and fluorescence microscopy

Excised tumors were fixed in 4% paraformaldehyde, paraffin-embedded, and sectioned at 4 µm thickness. Sections were stained with hematoxylin and eosin for morphological evaluation. Adjacent sections were imaged for mScarlet fluorescence with DAPI nuclear counterstaining. Imaging was performed on an Olympus VS200 slide scanner using standard DAPI and RFP channels. For quantification, 3-5 ROIs of 250 × 250 µm were analyzed. Fluorescent area was normalized to the total ROI and reported as the percent fluorescence-positive area per ROI.

### Bone marrow confocal microscopy

***In vivo* confocal microscopy of calvarial bone marrow.** Intravital confocal microscopy of the calvarial BM was performed to visualize expression of transgene in Lin⁻ cells in their native microenvironment. Imaging was conducted on a Zeiss LSM 880 laser-scanning confocal microscope mounted on an Axio Examiner.Z1 upright platform (Carl Zeiss) using a 20× water-dipping objective (NA 1.0, working distance 1.8 mm), providing a FOV of 425 × 425 μm at 512 × 512 pixel resolution. Mice were anesthetized and secured in a stereotaxic head holder. The scalp was shaved and a midline incision was made to expose the calvarium; double-distilled water (DDW) was applied to prevent dehydration during imaging. BM vasculature was labeled by retro-orbital injection (10-15 min prior to imaging) of CD31-FITC. For each mouse, 3-5 distinct calvarial regions of interest were imaged. Z-stacks were acquired with 4 μm step size to capture 3D marrow architecture.

***Ex vivo* confocal microscopy of femur.** After imaging, femurs were harvested, fixed in 4% PFA for 12-18 h, cryoprotected by incubation in 15% sucrose for 6 hours at 4 °C, embedded in OCT, and snap-frozen at -20°C. For fluorescence imaging, femurs were sectioned/shaved to a depth of 300 µm to expose the BM, using a cryostat set to -25 °C with a section thickness of 3-5 µm. Spinning-disk confocal microscopy was used to image mScarlet and DAPI signals and to visualize reporter-positive regions. Excitation wavelengths were **405** nm and 561 nm. Maximum-intensity projections were generated and processed in FIJI (ImageJ).

### Statistics

Statistical analyses were performed using GraphPad Prism (GraphPad Software). Data are presented as mean ± SEM. Normality was assessed using the Shapiro-Wilk test. Most datasets were nonparametric. Comparisons between two independent groups were performed using an unpaired two-tailed Student’s *t*-test for parametric data or the Mann-Whitney test for nonparametric data. For paired comparisons of contralateral tumors within the same mouse, the Wilcoxon matched-pairs signed-rank test was used. Comparisons among three independent groups were performed using one-way ANOVA followed by Tukey’s multiple-comparisons test for parametric data, or the Kruskal-Wallis test followed by Dunn’s multiple-comparisons test for nonparametric data. Cross-modal correlation analyses were performed at the individual-animal level using Spearman’s rank correlation coefficient (ρ) with pairwise available observations. Two-sided P values and 95% percentile bootstrap confidence intervals were calculated for each pairwise correlation. P < 0.05 was considered nominally significant; correlations with bootstrap confidence intervals spanning zero were interpreted as exploratory. The statistical test used and the number of animals or samples for each analysis are indicated in the corresponding figure legends.

## Results

### Dual CEST-fluorescent reporter design and efficient expression in CT26 colon carcinoma cells

To validate reporter expression, CT26 cells were transduced with the dual mScarlet-SC construct or the tdTomato control, enabling constitutive co-expression of the MRI reporter and red fluorescence from a single transcript (**Figure 1A**). Lentiviral particles were generated in HEK293T packaging cells, in which brightfield and fluorescence microscopy demonstrated robust reporter-associated signal, confirming transfection and reporter expression in packaging cells (**Figure S1**). After lentiviral transduction of CT26 cells (**Figure 1B**), both tdTomato- and mScarlet-SC-transduced cells exhibited strong red fluorescence relative to non-transduced controls (**Figure 1C**). Quantification of fluorescence micrographs as the fraction of red-fluorescent cells per region of interest (ROI) confirmed efficient reporter expression in CT26 cells (**Figure 1D**).

### Establishing *in vivo* in bilateral CT26 flank tumors by IVIS and CEST-MRI

We next evaluated the mScarlet-SC reporter in a preclinical *in vivo* setting to assess its potential for noninvasive imaging. Non-transduced CT26 cells and mScarlet-SC reporter-transduced CT26 cells were implanted into opposite flanks of the same BALB/c mouse (**Figure 2A**). *In vivo* optical imaging at 3 weeks post-implantation showed a higher mScarlet signal in reporter tumors than in contralateral control tumors (**Figure 2B**). CEST-MRI was then performed to evaluate deep-tissue reporter detectability. Tumor ROIs were used to generate CEST contrast maps (aka MTR_asym_ maps) overlaid on anatomical T2-weighted images (**Figure 2C**) and to extract ROI-averaged MTR_asym_ spectra (**Figure 2D**). Reporter-transduced tumors showed increased reporter-associated MTR_asym_ over the selected 1-2 ppm interval. Integrated CEST-MRI contrast, quantified as the MTR_asym_ area under the curve (AUC) over 1-2 ppm, was higher in mScarlet-SC reporter tumors than in contralateral controls (**Figure 2E**). This group difference is consistent with a reporter-associated CEST effect superimposed on the endogenous tissue background 17,18.

### *Ex vivo* optical imaging and histological analysis confirm tumor reporter expression

To verify that the *in vivo* imaging contrast reflected reporter expression, tumors were excised and analyzed *ex vivo*. Optical imaging showed higher mScarlet fluorescence in reporter tumors than in control tumors (**Figure 3A**). Quantification of fluorescence microscopy, expressed as the fraction of red-fluorescent cells, confirmed significantly greater reporter expression in mScarlet-SC tumors than in controls (**Figure 3B**). Consistent with these findings, representative histological images demonstrated robust mScarlet fluorescence in reporter tumors with DAPI nuclear counterstaining, together with corresponding morphology in H&E-stained sections (**Figure 3C**). Some morphological differences were evident between representative H&E sections, consistent with the expected intratumoral heterogeneity of CT26 tumors.

### Reporter expression in primary hematopoietic stem and progenitor cells

We next assessed whether the dual reporter could be expressed in biologically sensitive primary hematopoietic populations. Hematopoietic Lin⁻ stem and progenitor cells and/or LSK stem and progenitor cells were isolated from donor BM and transduced with reporter or control constructs as outlined in **Figure 4A**. Flow cytometry gating was used to define the Lin⁻, LSK, and LK populations for cell isolation and downstream analysis (**Figure 4B**; **Figure S4**). Red fluorescence microscopy confirmed a detectable reporter signal in Lin⁻ BM cells, with non-transduced cells serving as the negative baseline and tdTomato-transduced cells as a technical fluorescent positive control. Microscopy-based quantification showed higher fluorescence in reporter-transduced cells than in the non-transduced baseline (**Figure 4C-D**). Imaging flow cytometry (ImageStream) further resolved reporter expression at the single-cell level of LSK cells, providing fluorescence intensity distributions, representative cell images (**Figure 4E-F**), and quantitative single-cell fluorescence measurements and maintenance relative to the non-transduced baseline (**Figure 4G; Figure S6**). Together, these data show that the mScarlet-SC reporter can be expressed in primary Lin⁻ and LSK cells and that it supports quantitative fluorescence-based analysis in these populations. As an independent feasibility control for genetic manipulation of HSPCs, LSK cells were transduced with an eGFP-expressing lentiviral construct and labeled with a membrane dye prior to transplantation (**Figure S2A**). One week after injection, membrane dye-positive cells were detected in femoral BM (**Figure S2B**), consistent with short-term marrow localization of dye-labeled cells. These findings are consistent with previous studies showing that lentivirally transduced murine Lin⁻ and LSK cells can retain hematopoietic repopulating capacity 25.

### *In vivo* multimodal imaging demonstrates detectability of reporter-transduced Lin⁻ cells after engraftment

To evaluate reporter performance in a transplantation setting, mice underwent half-body irradiation followed by transplantation of reporter-transduced BM Lin⁻ cells and an 8-week engraftment period prior to multimodal imaging (**Figure 5A**). Whole-body IVIS imaging at 8 weeks revealed localized RFP signal on the irradiated/reconstituted side, consistent with engraftment and reporter retention, and quantification confirmed increased radiant efficiency in the reporter group (**Figure 5B-C**).

To assess deep-tissue detection within BM, femoral CEST-MRI was performed in non-irradiated controls, irradiated recipients transplanted with non-reporter-transduced Lin⁻ cells, and irradiated recipients transplanted with mScarlet-SC reporter-transduced Lin⁻ cells. T2-weighted anatomical images with overlaid MTR_asym_ maps demonstrated increased reporter-associated contrast within femoral marrow regions in the reporter-transduced group (**Figure 5D**), supported by the corresponding marrow MTR spectra (**Figure 5E**). The 0-1 ppm region exhibited marked differences, suggesting substantial contributions from non-reporter-related background in this spectral range. Quantitative integration was therefore restricted to the selected 1-2 ppm interval, where the reporter-associated group difference remained detectable. Integrated CEST contrast, quantified as the MTR_asym_ AUC over the 1-2 ppm interval, differed significantly among the three groups (**Figure 5F**). Reporter-transduced irradiated marrow showed higher MTR_asym_ AUC than both irradiated marrow reconstituted with non-reporter-transduced Lin⁻ cells and non-irradiated femoral marrow. These findings support an additional reporter-associated contribution to the measured femoral CEST contrast beyond baseline marrow signal and changes associated with irradiation and transplantation.

As a within-scan spatial control, CEST-MRI was additionally analyzed in the adjacent medial thigh adductor muscle compartment using the same processing pipeline and 1-2 ppm integration window (**Figure S3**). Muscle MTR_asym_ values were low, with AUC values centered near zero, and no significant differences were detected among non-irradiated controls, irradiated recipients transplanted with non-reporter-transduced Lin⁻ cells, and irradiated recipients transplanted with reporter-transduced Lin⁻ cells. The absence of a corresponding group-dependent change in adjacent muscle supports the spatial localization of the increased reporter-associated CEST contrast to the femoral marrow rather than a global scan-level shift. As an orthogonal *in vivo* validation, intravital calvarial microscopy visualized mScarlet⁺ marrow cells within BM niches 8 weeks after transplantation of reporter-transduced BM-derived Lin⁻ cells. The BM vasculature was labeled with CD31-FITC and merged images showed the spatial relationship between mScarlet⁺ cells and vascular structures (**Figure 5G**).

### *Ex vivo* validation confirms reporter retention in reconstituted hematopoietic compartments

To corroborate the *in vivo* imaging findings, BM was analyzed *ex vivo* 8 weeks after irradiation and transplantation. Confocal microscopy of femoral marrow demonstrated robust reporter-associated signal in mice reconstituted with mScarlet-SC reporter-transduced Lin⁻ cells compared with non-transduced Lin⁻ controls. Whole-region images acquired with DAPI nuclear staining and the red fluorescence channel revealed discrete reporter-positive regions throughout the marrow in transduced recipients (**Figure 6A**), which were also evident at higher magnification (**Figure 6B**). Quantification of fluorescence microscopy, expressed as the fraction of red-fluorescent cells per FOV, confirmed greater reporter-associated signal in mScarlet-SC BM than in controls (**Figure 6C**). At the organ level, *ex vivo* optical imaging of reconstituted tibias showed higher fluorescence signal in reporter-transduced recipients than in controls, and quantitative analysis confirmed increased tibial fluorescence in the reporter group (**Figure 6D-E**).

Flow cytometry provided complementary cellular-level validation of reporter retention within hematopoietic compartments. Representative plots showed mScarlet-positive events in reconstituted marrow, including within the Lin⁻ and LSK gates, in reporter-transduced recipients, whereas control samples showed minimal background signal (**Figure 6F; Figure S4**). Quantitative analysis further showed a higher percentage of mScarlet-positive cells within both the Lin⁻ and LSK populations in reporter-transduced recipients compared with non-labeled controls (**Figure 6G**). Additional analysis showed a positive association between expected and detected numbers of reporter-positive cells in both the Lin⁻ and LSK populations (**Figure S5**). Because the CEST-MRI voxels encompass the whole femoral marrow region, the 8-week CEST signal should be interpreted as the aggregate signal from reporter-positive donor-derived hematopoietic cells and their differentiated progeny, rather than as a selective measurement of the primitive HSPC niche. The Lin⁻ and LSK flow-cytometry results confirm reporter presence in phenotypically defined compartments but do not establish that the MRI signal originates predominantly from primitive HSPCs. To further provide an exploratory estimate of the reporter-positive cell burden associated with femoral CEST contrast, we combined total femoral bone-marrow cellularity with a confocal-derived mScarlet-positive fraction estimate from five reporter-transduced recipients. The MRI-derived femoral marrow volume corresponded to approximately 38.4 CEST-voxel equivalents per femur, based on a CEST voxel volume of 0.391 μL. The estimated reporter-positive burden ranged from 4.66 × 10⁴ to 1.61 × 10⁵ cells per marrow-equivalent CEST voxel (**Table S1**). Across these five animals, the estimated burden was not significantly associated with femoral MTRasym AUC over 1-2 ppm (Spearman ρ = 0.10, P = 0.873, n = 5). These values provide an exploratory order-of-magnitude estimate under the present experimental conditions and do not establish a formal detection threshold or a calibrated cell-to-signal relationship.

Finally, several exploratory pairwise associations among cellular and imaging readouts met both the nominal P-value criterion and the bootstrap confidence-interval criterion (Figure 6H; Table S2). Comparisons with bootstrap confidence intervals spanning zero were treated as exploratory and are shown without significance symbols. Together, these data support cross-modal concordance between *in vivo* imaging and *ex vivo* measures of reporter retention without implying that the different modalities provide interchangeable quantitative measures of reporter-positive cell burden.

## Discussion

Using a stepwise validation strategy, this study demonstrates that the dual mScarlet-SC reporter is expressed efficiently in both CT26 cells and primary hematopoietic populations, generates measurable pre-clinical *in vivo* optical and CEST-MRI contrast, and remains detectable after transplantation in mice with reconstituted deep BM. Tumor studies established reporter performance under controlled within-animal conditions, whereas transplantation studies showed multimodal detectability of reporter-transduced Lin⁻ cells in marrow by IVIS, femoral CEST-MRI, and intravital microscopy, with *ex vivo* confirmation by confocal imaging and flow cytometry. Together, these findings support the feasibility of this dual-reporter platform for cross-validated imaging of reporter-transduced hematopoietic cells in deep BM.

Among genetically encoded MRI reporter strategies, intrinsically CEST-active proton-exchange reporters offer several conceptual advantages. Unlike ferritin-based reporters, they do not depend on the intracellular accumulation of paramagnetic iron 26. Unlike transporter-based reporters such as Oatp1a1 and Oatp1b3, intrinsically CEST-active polypeptides do not require administration of an exogenous contrast substrate at the time of imaging 27,28. Instead, their contrast is generated by applying a frequency-selective saturation pulse, making the reporter technically compatible with repeated measurements without contrast-agent injection. This substrate-free advantage does not apply to every genetically encoded CEST system. The dual-color GeneREFORM system requires the administration of synthetic nucleoside reporter probes 29, whereas genetically encoded HyperCEST reporters require hyperpolarised ^129^Xe and specialized acquisition methods 30.

The proton-exchange CEST-reporter design space includes several distinct polypeptide architectures. The original synthetic lysine-rich protein, LRP, concentrated exchangeable backbone amide protons but contained a highly repetitive coding sequence 12. The redesigned lysine-rich protein, rdLRP, reduced DNA repetitiveness and incorporated lysine-separated arginine-, histidine-, glycine-, and proline-containing motifs to improve genetic stability while retaining proton-exchange contrast 14. Human protamine-1, hPRM1, provided a compact human-derived, arginine-rich reporter with detectable guanidyl- and amide-associated CEST effects 31, whereas supercharged GFP variants combined proton-exchange CEST contrast with intrinsic fluorescence in a single protein 32. More recently, an AAV-delivered CEST polypeptide comprising 150 arginine residues fused to tdTomato was developed for longitudinal imaging of neuronal transgene expression, with spectral features reported near 0.75 and 1.75 ppm 33. Mechanistically distinct approaches include probe-dependent dual-color enzyme reporters 29 and gas-vesicle HyperCEST reporters detected using hyperpolarised ^129^Xe 30. Within this broader landscape, SuperCESTide was computationally assembled from multiple CEST-active peptide motifs to increase amino acid diversity and reduce highly repetitive sequences 17. Compared with earlier repetitive polypeptide reporters, this design may improve genetic flexibility, stability, and cellular compatibility while maintaining detectable reporter-associated CEST contrast. These properties provided the rationale for evaluating SuperCESTide in sensitive primary hematopoietic cells and for combining it with mScarlet-I to enable orthogonal cellular validation.

Building on this design, recent studies have established the feasibility of SuperCESTide imaging *in vivo* 17,18. In particular, rAAV-mediated delivery of SuperCESTide to the liver demonstrated that reporter-associated CEST contrast can be detected in deep tissue and highlighted the value of orthogonal fluorescence-based validation 18. Extending SuperCESTide imaging from hepatocytes to HSPCs in bone marrow, however, presents a substantially greater challenge. The prior *in vivo* demonstration was performed in hepatocytes, which are large, metabolically active cells and constitute the predominant parenchymal cell population within the imaged organ 18. In contrast, reporter-transduced Lin⁻ hematopoietic cells are smaller, less abundant, and distributed throughout a heterogeneous marrow microenvironment containing adipocytes, stromal cells, and endogenous hematopoietic populations. Together, these features are expected to reduce the effective contrast-to-noise ratio and make reporter detection more demanding.

Our study both complements and extends this earlier work in several important respects. Rather than imaging transgene expression in a parenchymal organ after systemic vector delivery, we use the reporter in transplanted cells that must withstand *ex vivo* manipulation, home to the marrow, and persist within a specialized niche. We also implement the reporter in a lentiviral dual-reporter cassette that is directly compatible with engineered-cell applications. Finally, we establish a cross-modal validation framework in which optical imaging, intravital microscopy, MRI, and *ex vivo* cellular assays converge on the same biological endpoint. Taken together, these findings move SuperCESTide beyond a proof-of-expression setting and support its use as a cross-validated imaging tool for reporter-transduced Lin⁻ hematopoietic cells *in vivo*.

A particularly important consideration in the present study is CEST signal specificity. CEST-MRI is not inherently reporter-exclusive because *in vivo* contrast can be influenced by endogenous exchangeable proton pools, direct water saturation, relayed nuclear Overhauser effects, semisolid magnetization-transfer asymmetry, local tissue composition, and B₀/B₁ inhomogeneities 34-38. We therefore designed the study around matched controls and orthogonal validation rather than relying on MRI alone. Several features strengthen the interpretation of the BM signal, including WASSR-based voxel-wise B_0_ correction 34, ROI-based analysis within defined marrow regions, comparison with non-transduced transplanted controls, comparison with non-irradiated femoral marrow, and independent confirmation of reporter retention by fluorescence imaging, intravital microscopy, confocal microscopy, and flow cytometry. The finding that irradiated marrow transplanted with reporter-transduced cells showed higher CEST contrast than both non-transduced transplanted marrow and non-irradiated marrow supports the interpretation that the observed group difference is not explained solely by baseline marrow asymmetry or irradiation-induced changes. An irradiated PBS-only sham group was not included, so irradiation-related and other microenvironmental effects cannot be fully separated. Irradiation-related hypocellularity, vascular permeability, edema, and adipose replacement may alter the free-water fraction and tissue T1/T2 relaxation properties. Loss or remodeling of hematopoietic, stromal, and extracellular-matrix components may also change the size and exchange properties of mobile and semisolid macromolecular pools, thereby affecting direct water saturation, magnetization transfer, relayed nuclear Overhauser effects, and apparent CEST contributions across the Z-spectrum. Consequently, the measured 1-2 ppm AUC represents a group-level contrast difference under the specific irradiation and reconstitution conditions and should not be interpreted as independent of tissue state. However, reporter- and non-transduced recipients underwent identical irradiation, transplantation, and follow-up. Thus, the non-transduced group provided the most relevant control, while non-irradiated mice provided a baseline reference.

*In vivo*, reporter-transduced tissues exhibited a broad downfield MTRasym elevation rather than a narrow, consistently resolved peak, with an apparent trend toward increased signal around 1.8 ppm and some inter-animal variability in the detailed spectral shape. SuperCESTide is an engineered protein assembled from multiple peptide sequences and contains diverse amino-acid compositions that may contribute overlapping exchangeable proton pools 17. The observed response is therefore more appropriately interpreted as a broad reporter-associated spectral effect superimposed on endogenous tissue background. The 0-1 ppm region showed marked differences, particularly between irradiated and non-irradiated marrow. Because this region is close to the direct water-saturation line and is sensitive to residual B₀ misregistration, spillover, macromolecular effects, and conditioning-related changes in marrow composition, it was excluded from the primary AUC analysis. Quantitative integration was therefore restricted to the 1-2 ppm interval as a more conservative reporter-weighted range. This interval remains subject to endogenous background and should not be interpreted as spectrally unique to SuperCESTide.

Signal within the 1-2 ppm interval may include contributions from reporter-derived exchangeable protons, endogenous amine and hydroxyl pools, semisolid magnetization-transfer asymmetry, relayed nuclear Overhauser effects, direct water saturation, and local tissue composition 35,36. Conventional MTRasym cannot completely separate these components because the positive-offset signal is referenced to the corresponding upfield offset, which may itself contain asymmetric MT or rNOE contributions. In principle, multipool Lorentzian fitting or Bloch-McConnell modeling could estimate reporter-associated exchange rates and proton-pool amplitudes separately from endogenous MT and rNOE components; however, the current single-power MTRasym dataset does not support reliable spectral deconvolution. Accordingly, the 1-2 ppm AUC should be interpreted as a reporter-associated group difference supported by matched controls and orthogonal validation, rather than as a chemically isolated SuperCESTide signal. Future studies incorporating B₁ mapping, saturation-power dependence, multipool Lorentzian analysis, Bloch–McConnell modeling, and asymmetry-independent metrics will be needed to separate reporter-derived and endogenous signal components more rigorously 35-38.

A related consideration is the sensitivity of CEST-MRI. *In vitro* reporter measurements cannot be directly extrapolated to an *in vivo* cell-number detection limit because endogenous CEST and magnetization-transfer background, tissue composition, cellular distribution, partial-volume effects, B₀/B₁ homogeneity, and acquisition conditions vary across tissue microenvironments 39. Using femur-normalized MRI volume and confocal mScarlet estimates, reporter-positive burden was 4.66 × 10⁴-1.61 × 10⁵ cell equivalents per CEST voxel (**Table S1**). This estimate was not associated with femoral MTRasym AUC and does not establish a detection threshold or calibrated cell-to-signal relationship. Perlman *et al*. 14 reported increased CEST contrast after implanting 1 × 10⁵ rdLRP-expressing GL261N4 cells, a comparable order of magnitude. The cross-modal correlations shown in **Figure 6H** address a separate question and were calculated across reporter-transduced and control animals. Future studies using controlled cell-dose titration, larger cohorts, repeated imaging, and improved B₀/B₁ correction will be required to define the *in vivo* detection limit.

Several limitations should be acknowledged. IVIS provides quantitative fluorescence measurements, but tissue scattering, absorption, and depth make its signal a less direct measure of cellular burden, particularly in bone; it was therefore interpreted primarily as a validation readout. The transplantation studies were designed as a feasibility study at a single late time point and were not intended to quantify absolute donor-cell burden or reporter distribution across all marrow-containing skeletal sites. Endpoint flow cytometry confirmed mScarlet⁺ cells in the Lin⁻ and LSK compartments but could not distinguish the originally transplanted reporter-positive cells from their reporter-positive progeny; the separate membrane dye experiment assessed only 7-day homing, not the 8-week CEST signal. The femoral CEST-MRI resolution does not permit single-cell localization, and partial-volume contributions from cortical bone, surrounding tissue, and heterogeneous marrow cannot be excluded. Micro-CT/MRI co-registration was not performed in the present study. Although ROI placement was guided by high-resolution T2-weighted images and supported by matched controls, future higher-resolution imaging and anatomical co-registration will be needed for voxel-level spatial validation. Although numerous mScarlet-positive cells remained detectable in bone marrow at 8 weeks, indicating persistence of reporter-expressing cells, partial immune-mediated clearance or transgene silencing cannot be excluded. Moreover, reporter-positive cells persisted within phenotypically defined LK and LSK compartments, suggesting compatibility with maintenance of hematopoietic progenitor phenotypes *in vivo*, although subtle effects on HSPC function cannot be excluded. However, viability, apoptosis, colony-forming capacity, and functional hematopoietic reconstitution were not directly assessed; dedicated functional studies will therefore be required to determine whether reporter expression affects HSPC fitness. Although T2A cleavage is generally efficient, a small residual uncleaved fraction cannot be excluded because it was not directly measured. The reporter was evaluated in murine hematopoietic progenitor populations and not in human CD34+ HSPCs. Therefore, the current data should be interpreted as a preclinical proof-of-concept rather than evidence of immediate clinical applicability. Future studies incorporating earlier serial imaging, donor-chimerism measurements, and complementary lineage-tracing approaches will help refine the relationship between reporter signal and transplanted cell burden.

For eventual translation, safety and immunogenicity will require careful consideration. Although the present study was designed as a preclinical discovery framework, clinical deployment of genetically encoded reporters faces challenges including long-term expression control, regulatory acceptability, and possible humoral or cellular immune responses against non-self reporter products, especially in HSPC-directed applications 4,6. The relatively compact size of SuperCESTide may facilitate cassette design, although whether this also reduces immunogenic liability remains unknown. In the near term, the principal value of the platform may be in preclinical optimization of gene and cell therapies through noninvasive confirmation of marrow localization and persistence. More broadly, these findings support multimodal reporter design for cell-therapy imaging: optical reporters provide efficient screening and *ex vivo* validation 40-42, whereas MRI provides deep-tissue access and the potential for longitudinal readout 11,12. Combining these strengths in a single cassette may be particularly useful for hematopoietic applications and potentially adaptable to other engineered-cell settings.

In conclusion, this study demonstrates that a dual mScarlet-SC reporter can support cross-validated, noninvasive detection of engineered cells in deep BM. By integrating deep-tissue CEST-MRI with fluorescence imaging, intravital microscopy, and *ex vivo* cellular analyses, the study provides a framework linking MRI contrast to verified reporter expression in hematopoietic compartments. These findings demonstrate the feasibility of imaging established hematopoietic engraftment and provide a foundation for future longitudinal studies. Future studies incorporating earlier and repeated imaging time points, quantitative measurements of donor-cell burden, and assessment of inter-session imaging reproducibility will be required to establish the platform as a longitudinal tool.

## Supplementary Material

Supplementary methods, figures and tables.

## Figures and Tables

**Figure 1:**
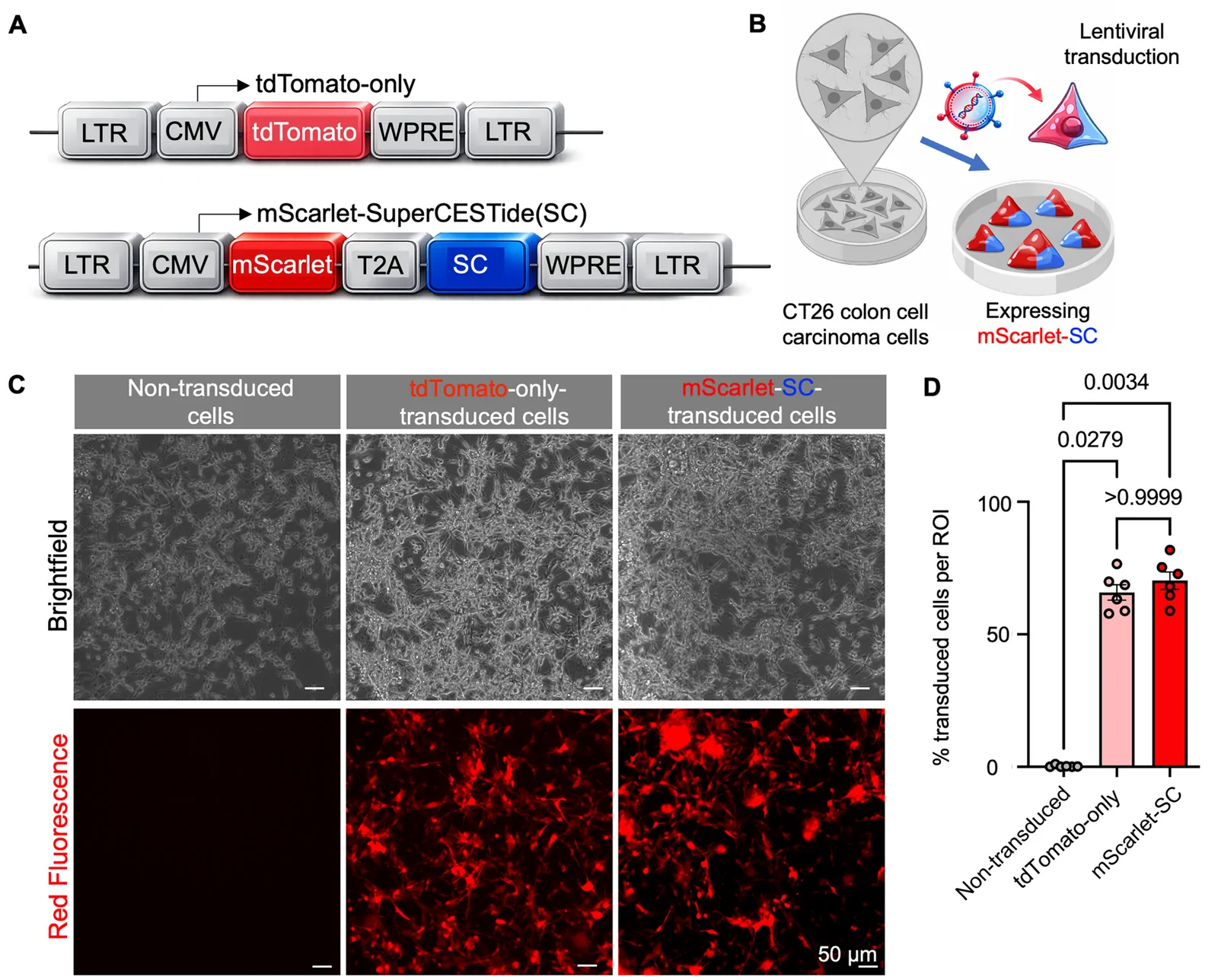
** Design of the dual CEST-fluorescent reporter and *in vitro* validation in CT26 colon carcinoma cells. (A)** Schematic of the lentiviral dual-reporter cassette encoding the red fluorescent protein mScarlet-I (hereafter mScarlet) linked via a T2A sequence to the CEST reporter SuperCESTide (SC), under the indicated promoter and lentiviral backbone elements. A fluorescent-only lentiviral control (tdTomato) is shown as a positive control for transduction and red fluorescence.** (B)** Overview of the *in vitro* CT26 lentiviral transduction workflow; cells were transduced and expanded under standard culture conditions.** (C)** Representative brightfield and fluorescence microscopy images of CT26 cells from three groups: (i) non-transduced cells, (ii) fluorescent-only control cells expressing tdTomato, and (iii) dual reporter-transduced cells expressing mScarlet-SC. No obvious morphological abnormalities were observed by brightfield microscopy.** (D)** Quantification of transduction efficiency in CT26 cells, expressed as the percentage of red fluorescent cells and calculated from 3 ROIs per condition across 6 independent experiments. (Each dot represents a separate experiment; P < 0.05 is significant; Kruskal-Wallis statistical test followed by Dunn’s test).

**Figure 2:**
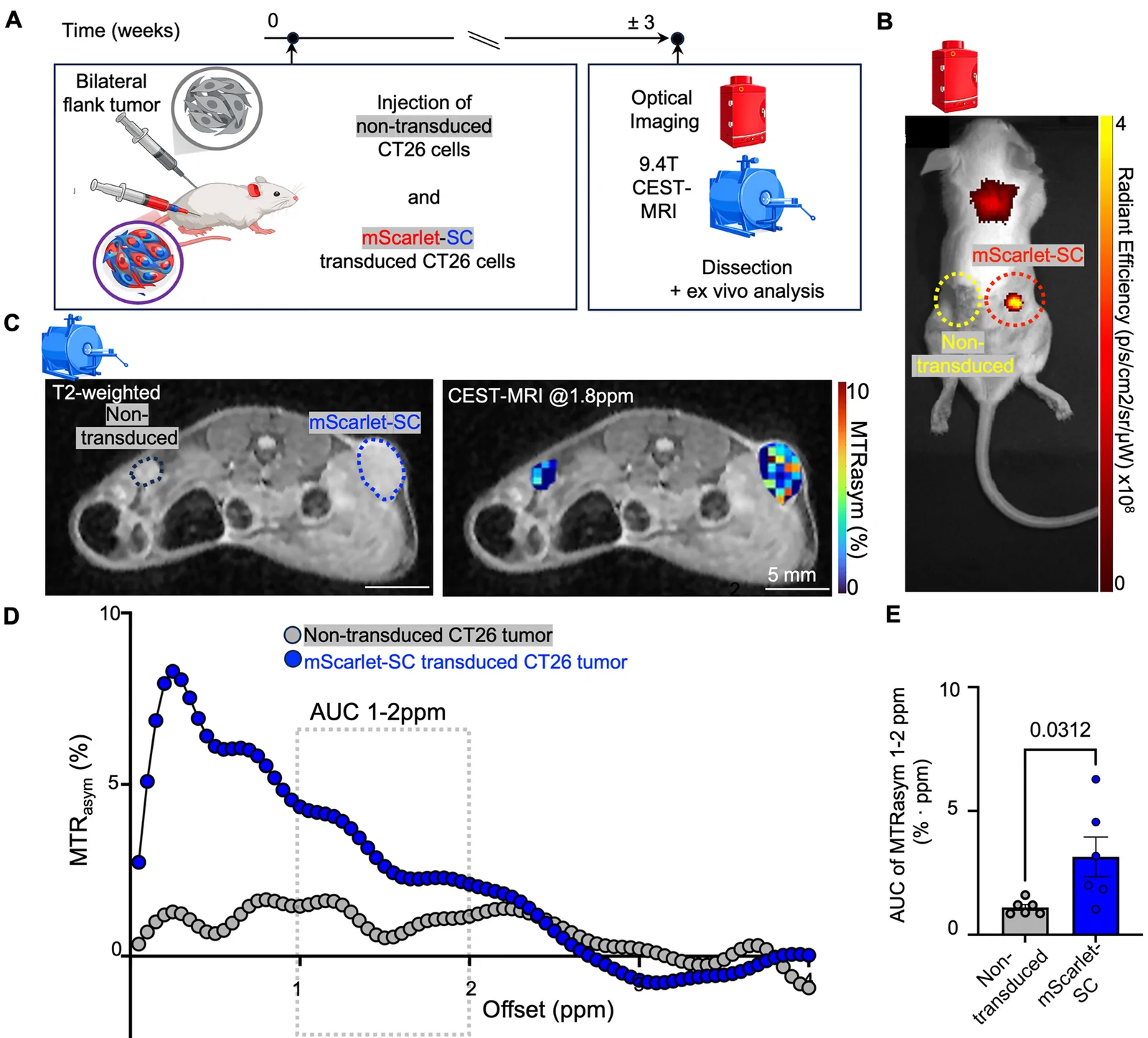
***In vivo* validation of the dual CEST-fluorescent reporter in CT26 cells. (A)** Experimental schematic of the bilateral flank tumor model in BALB/c mice, with non-transduced CT26 cells implanted in one flank and mScarlet-SC-transduced CT26 cells implanted contralaterally in the same animal. **(B)** Representative *in vivo* fluorescence imaging (IVIS) showing reporter-transduced and control tumors within the same mouse.** (C)** Representative *in vivo* MRI of flank tumors showing T2-weighted anatomical images with corresponding CEST-MRI MTR_asym_ maps at 1.8 ppm for control and reporter tumors.** (D)** MTR_asym_ spectra as a function of saturation frequency offset derived from tumor ROIs for control and contralateral reporter tumors (n = 6 mice, with one reporter-transduced and one non-transduced tumor per mouse; six paired comparisons).** (E)** Integrated CEST-MRI contrast quantified as the MTR_asym_ area under the curve (AUC) over 1-2 ppm (%·ppm) for control versus contralateral reporter tumors (n = 6 paired comparisons). (Each dot represents one tumor; Wilcoxon matched-pairs signed-rank statistical test).

**Figure 3:**
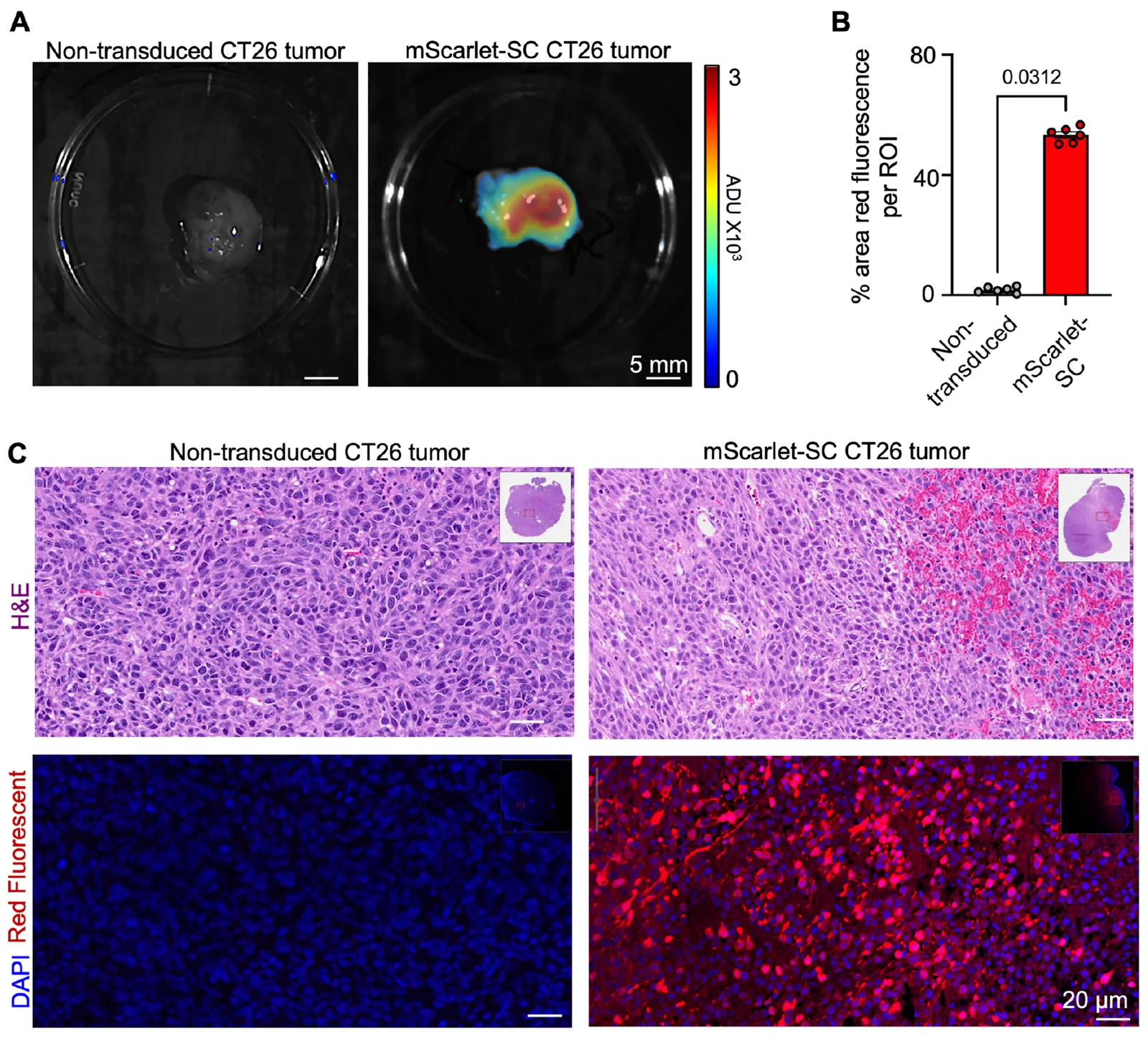
***Ex vivo* and histological validation of reporter expression in CT26 tumors. (A**) Representative *ex vivo* optical imaging of excised control and mScarlet-SC-expressing CT26 tumors.** (B)** Quantification of tumor fluorescence microscopy, reported as the fraction of red fluorescent cells per region of interest (ROI) in control and reporter tumors (n=6 per group). (Each dot represents a mouse; P < 0.05 is significant; Wilcoxon matched-pairs signed-rank statistical test) **(C)** Representative tumor histology and fluorescence microscopy. Paraffin sections from control and reporter tumors were stained with H&E (morphology) and imaged for mScarlet fluorescence with nuclear counterstain (DAPI) in adjacent sections.

**Figure 4:**
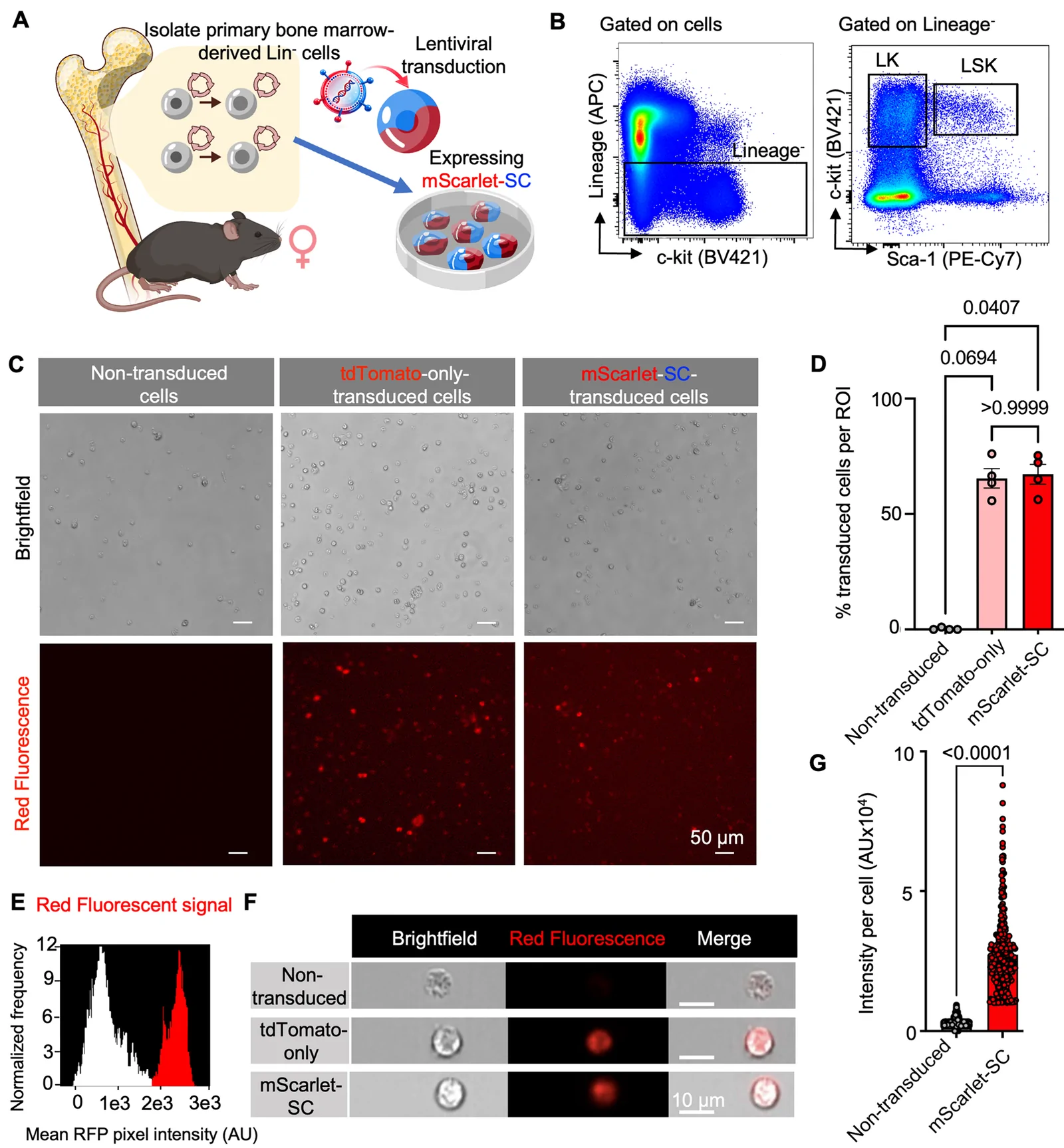
** Reporter expression in bone-marrow (BM)-derived hematopoietic progenitor cells. (A)** Workflow schematic showing BM isolation from female donor mice, enrichment and/or sorting of hematopoietic lineage-negative (Lin⁻) cells and Lin⁻Sca-1⁺c-Kit⁺ (LSK) cells, followed by lentiviral transduction with the mScarlet-SuperCESTide (SC) reporter or control constructs.** (B)** Flow cytometry gating strategy used to define the Lin⁻, LK, and LSK populations, with LK cells identified as Lin⁻c-Kit⁺ and LSK cells as Lin⁻Sca-1⁺c-Kit⁺.** (C)** Representative fluorescence microscopy images of transduced Lin⁻ cells from the indicated groups (mScarlet-SC reporter, tdTomato-only control, and non-transduced control).** (D)** Quantification of transduction efficiency in Lin⁻ cells, expressed as the percentage of RF⁺ cells in the indicated population (Each dot represents a separate experiment; one-way ANOVA followed by Tukey’s multiple-comparisons test).** (E)** ImageStream analysis showing the distribution of single-cell red fluorescence across conditions, presented as mean pixel intensity histograms. **(F)** Representative ImageStream images of non-transduced LSK cells, tdTomato-transduced and mScarlet-SC reporter cells.** (G)** Quantification of single-cell red fluorescence intensity (AU) by ImageStream for non-transduced LSK cells (n = 3958), and mScarlet-SC-transduced LSK cells (n = 291) (unpaired two-tailed Student’s *t*-test).

**Figure 5:**
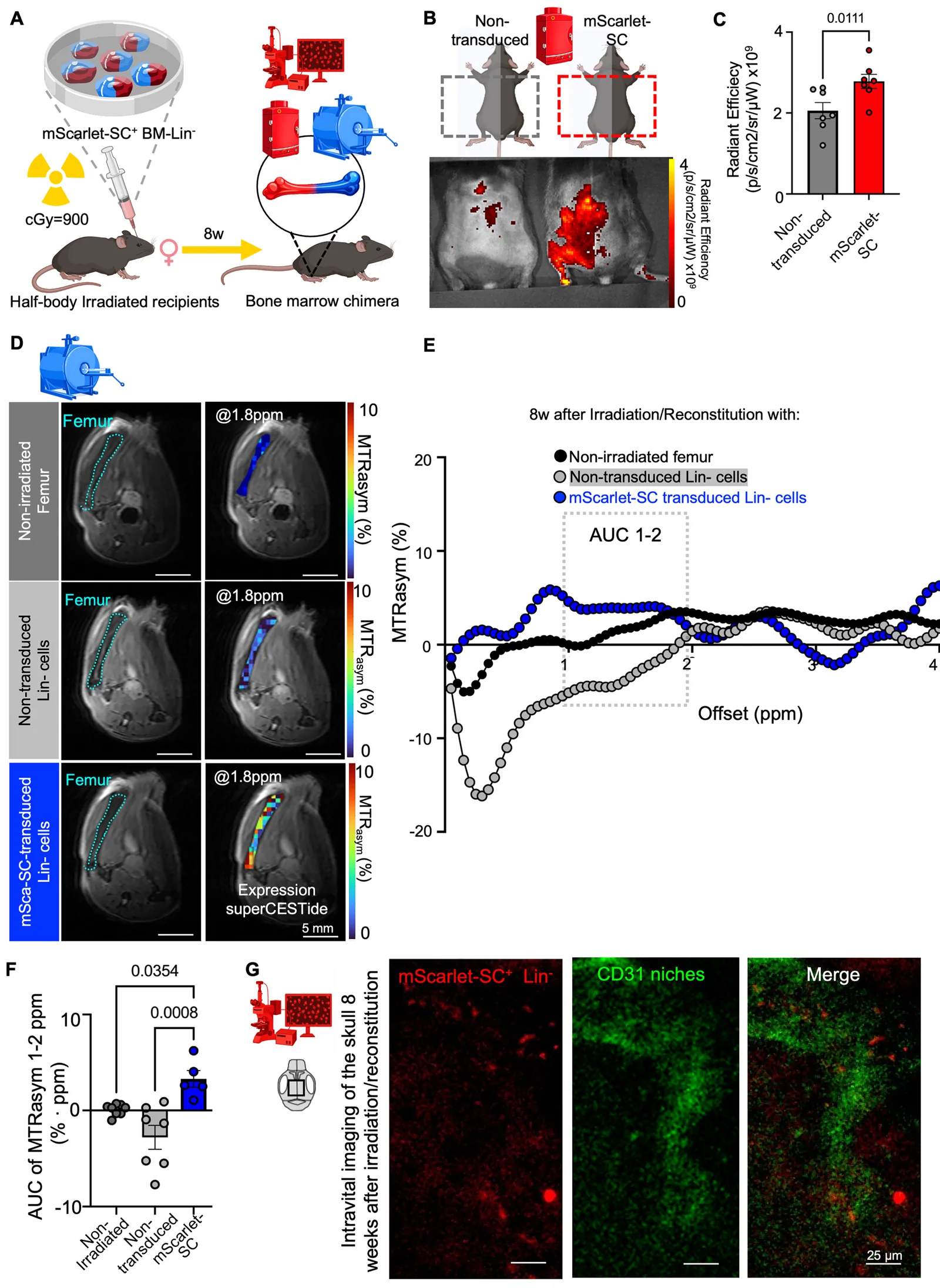
***In vivo* engraftment and multimodal tracking of mScarlet-SC-transduced Lin⁻ cells by optical imaging, CEST-MRI, and intravital microscopy. (A)** Schematic of the unilateral irradiation and transplantation protocol. Mice received bone-marrow-derived mScarlet-SC-transduced Lin⁻ cells after unilateral irradiation and were imaged 8 weeks later by whole-body IVIS, femoral CEST-MRI, and calvarial intravital microscopy. **(B)** Representative whole-body IVIS image showing localized mScarlet fluorescence on the irradiated/reconstituted side 8 weeks after transplantation. **(C)** Quantification of IVIS radiant efficiency (n = 7 per group; Mann–Whitney test). **(D)** Representative femoral T2-weighted anatomical images with overlaid MTRasym maps for non-irradiated controls, irradiated recipients transplanted with non-transduced Lin⁻ cells, and irradiated recipients transplanted with mScarlet-SC-transduced Lin⁻ cells. All MTRasym maps were processed identically and displayed using the same color scale. **(E)** Mean MTRasym spectra from femoral BM ROIs for non-irradiated controls (n = 8), irradiated recipients transplanted with non-transduced Lin⁻ cells (n = 7), and irradiated recipients transplanted with mScarlet-SC-transduced Lin⁻ cells (n = 5). **(F)** Integrated femoral CEST contrast, quantified as the MTRasym area under the curve (AUC) from 1-2 ppm (%·ppm), for non-irradiated controls (n = 8), irradiated recipients transplanted with non-transduced Lin⁻ cells (n = 7), and irradiated recipients transplanted with mScarlet-SC-transduced Lin⁻ cells (n = 5). Groups were compared using the Kruskal–Wallis test followed by Dunn’s multiple-comparisons test. **(G)** Representative calvarial intravital microscopy images showing mScarlet⁺ marrow cells and CD31-FITC-labeled vasculature, together with corresponding merged images, 8 weeks after transplantation of mScarlet-SC-transduced Lin⁻ cells.

**Figure 6:**
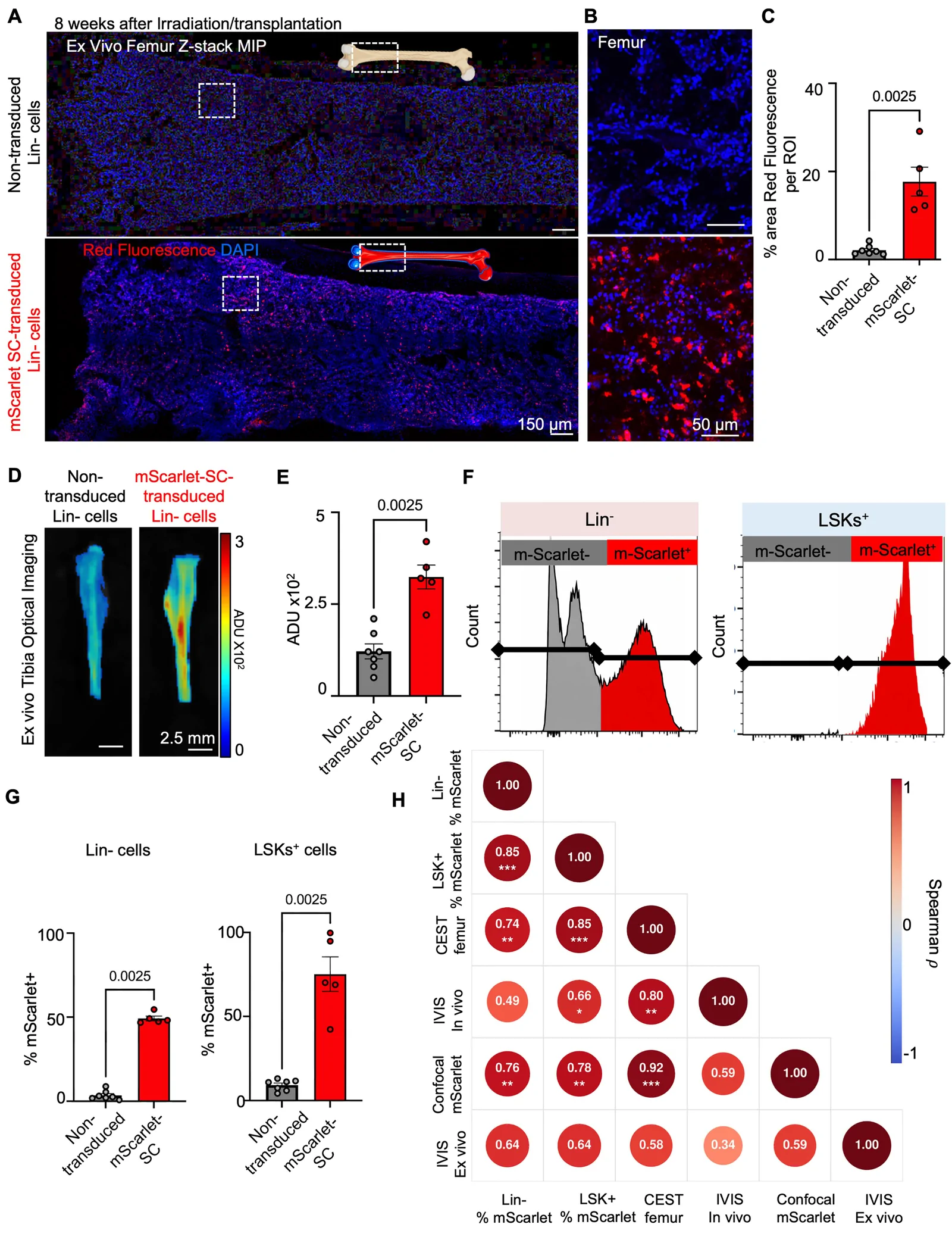
***Ex vivo* validation of reporter retention in reconstituted bone marrow (BM) 8 weeks after irradiation and transplantation by fluorescence imaging and flow cytometry. (A)** Representative *ex vivo* maximal intensity projections (MIP) of femoral BM from mice transplanted with non-labeled Lin⁻ cells or mScarlet-SC reporter-transduced Lin⁻ cells, shown as whole-region views with DAPI nuclear staining and red fluorescence confocal microscopy. **(B)** Higher-magnification confocal micrographs of marrow regions from the same groups.** (C)** Quantification of BM fluorescence microscopy, reported as the fraction of red fluorescent cells per FOV in control and reporter BM (non-transduced controls, n = 7; reporter-transduced, n = 5). (D) Representative *ex vivo* optical imaging of reconstituted tibias from non-labeled control and reporter-transduced recipients. **(E)** Quantification of *ex vivo* tibial fluorescence signal (control, n = 7; reporter-transduced, n = 5).** (F)** Representative flow cytometry plots showing mScarlet signal within reconstituted BM, including the Lin⁻ and LSK compartments, in non-labeled control and reporter-transduced recipients (non-transduced controls, n = 7; reporter-transduced, n = 5). **(G)** Percentage of mScarlet⁺ cells within the Lin⁻ and LSK populations. **(H)** Exploratory cross-modal Spearman correlation matrix showing pairwise associations among mScarlet⁺ Lin⁻ and LSK cells, femoral CEST-MRI MTRasym AUC over 1-2 ppm, *in vivo* IVIS fluorescence, femoral confocal mScarlet-positive area, and *ex vivo* tibial IVIS fluorescence. Pairwise complete observations were used (n = 12 for all comparisons). Circle size and color indicate the magnitude of Spearman’s ρ, with ρ values shown to two decimal places. Asterisks indicate associations meeting both criteria of P < 0.05 and a 95% percentile bootstrap confidence interval excluding zero: *P < 0.05, **P < 0.01, and ***P < 0.001. Comparisons with confidence intervals spanning zero are shown without asterisks. Pairwise sample sizes and confidence intervals are provided in **Table S2**. For quantitative panels C, E, and G, each dot represents one mouse; group comparisons used the Mann–Whitney U test.

## Data Availability

All data supporting the findings of this study are included in the manuscript and supplementary material. Additional raw datasets are available from the corresponding author upon reasonable request.
