# Supplementary material for: A dual CEST-fluorescent reporter enables cross-validated tracking of hematopoietic engraftment in deep bone marrow
Source: Theranostics. 2026 Sep 24;16(16):9416–33. doi: 10.7150/thno.140697 (PMC13684844; doi:10.7150/thno.140697)
Supplement: Supplementary file 1 [file thnov16p9416s1.pdf]

Supplementary Material to:

A Dual CEST-Fluorescent Reporter Enables Cross-Validated Tracking of Hematopoietic Engraftment in Deep Bone Marrow

Tehila Alter[1], Narmeen Haj[1], Doaa Ali-Naffaa[1], Maya Rom[1], Ramez Daniel[1], Galit Saar[2], Assaf A. Gilad[3,4], and Katrien Vandoorne[1]*

AFFILIATIONS:

[1] Faculty of Biomedical Engineering, Technion-Israel Institute of Technology, Haifa, Israel

[2] Biomedical Core Facility, Rappaport Faculty of Medicine, Technion-Israel Institute of Technology, Haifa, Israel

[3] Biomedical Engineering Faculty, Tel Aviv University, Tel Aviv, Israel

[4] Chemical Engineering and Radiology, Michigan State University, East Lansing, USA

*Corresponding Author:

Katrien Vandoorne, DVM PhD

Associate Professor of Biomedical Engineering

Silver Building, Room 355

Technion - Israel Institute of Technology

Email: k.vandoorne@technion.ac.il

Supplementary Methods

Membrane dye labeling, lentiviral transduction, cell sorting, and transplantation of BM-derived LSK cells. Bone-marrow-derived lineage-negative Sca-1$^+$ c-Kit$^+$ (LSK) cells were isolated from male C57BL/6J donor mice. For cultured groups, bone marrow cells were enriched for LSKs using magnetic bead-based isolation (Miltenyi MACS), followed by culture and/or lentiviral transduction as described below. LSK cells were sorted using a BIGFOOT Spectral Cell Sorter (Invitrogen) conducted within the instrument's integrated Class II biocontainment cabinet. Data acquisition and sort setup were controlled using Sasquatch Software (SQ). Samples and collection tubes were maintained at 4 °C during sorting, and sorted cells were collected into 1.5 mL tubes.

After sorting, LSK cells were transduced with lentiviral particles generated from a pLenti_CMV_eGFP plasmid. For lentiviral transduction, $1x10^6$ LSK cells were plated in a 12-well plate and incubated in F-12 medium supplemented with 6 µg/mL polybrene, together with pLenti_CMV_eGFP lentiviral supernatant, for approximately 25 hours. Control cells were cultured under identical conditions in 1 mL F-12 medium containing 6 µg/mL polybrene, but without viral vector. Where indicated, LSK cells were processed and transduced similarly to Lin$^-$ cells.

After culture or transduction, cells were labeled with Cytopainter membrane dye (ab219942, Abcam) according to the manufacturer's instructions to enable short-term tracking after transplantation. To assess short-term persistence and marrow localization after ex vivo manipulation, membrane-labeled cells were transplanted into recipient mice. For transplantation, $2.5x10^4$ LSK cells per mouse were resuspended in sterile PBS and administered by retro-orbital injection into male C57BL/6J recipient mice. The Supplementary short-term homing experiment was performed separately using male C57BL/6J donor and recipient mice; the principal mScarlet-SC transplantation study used female C57BL/6J mice.

Experimental design. BM-LSK cells were isolated from male C57BL/6J donor mice and assigned to two experimental groups. The groups were as follows:

1. **Non-transduced LSKs:** non-transduced LSK cells cultured in F-12 medium with polybrene (6 µg/mL).

2. **Transduced LSKs:** LSK cells cultured in F-12 medium containing pLenti_CMV_eGFP lentiviral supernatant and polybrene (6 µg/mL) for approximately 25 hours.

All groups were labeled with membrane dye before transplantation into male C57BL/6J recipient mice. In vivo homing was evaluated 24 h after transplantation using intravital confocal microscopy of the calvarium, and bone marrow localization was subsequently confirmed 6 days later by ex vivo confocal microscopy of femoral cryosections obtained after sacrifice. This separate eGFP/membrane dye experiment assessed only short-term homing and was not used to evaluate 8-week reporter persistence or CEST-MRI signal. The 8-week signal may represent surviving reporter-positive donor cells and/or their hematopoietic progeny; the study design does not distinguish the initially transplanted founder HSPCs from their descendants.

Ex vivo validation using femur cryosections. To validate in vivo findings and assess the localization of transplanted cells within the femoral bone marrow, femurs were collected after transplantation, processed for cryosectioning, and analyzed by spinning disk confocal microscopy. Four-channel imaging of the membrane dye, eGFP, CD31, and DAPI was used to visualize transplanted cells and the bone-marrow vasculature.

Both control and eGFP-transduced LSK cells were detected in bone marrow sections, with membrane-labeled cells distributed throughout the tissue.

Estimation of expected reporter-positive Lin⁻ and LSK cell numbers

Expected reporter-positive Lin⁻ and LSK cell numbers were estimated from the total number of viable cells recovered from each tibia. Based on the expected cellular composition, Lin⁻ cells were assumed to represent approximately 30% of viable bone-marrow cells. The expected number of reporter-positive Lin⁻ cells was then calculated by multiplying the estimated number of Lin⁻ cells by 0.60, corresponding to an estimated 60% transduction efficiency. To estimate the expected number of reporter-positive LSK cells, the expected reporter-positive Lin⁻ cell number was further multiplied by 0.018, representing the expected proportion of LSK cells within the Lin⁻ population. Expected values were subsequently compared with the corresponding reporter-positive Lin⁻ and LSK cell numbers detected by ex vivo flow cytometry.

Expected and detected reporter-positive cell numbers were compared separately for Lin⁻ and LSK populations. Associations between expected and detected cell numbers were evaluated within the non-transduced and mScarlet-SC-transduced groups using Spearman's rank correlation coefficient. Average ranks were assigned in the presence of ties, and exact two-sided p values were calculated by permutation of all possible ranked observations. To assess relative detection, detected cell numbers were normalized to the corresponding expected values and expressed as a percentage of the expected. Differences between non-transduced and mScarlet-SC-transduced samples were evaluated using an exact two-sided Mann-Whitney U test based on all possible group allocations. For each mouse, only the left tibial bone marrow sample was included in the analysis. Data are presented as individual observations, with the median and interquartile range (IQR) where applicable. Statistical analyses were performed using GraphPad Prism, except for the expected-versus-detected cell-number analysis shown in Supplementary Figure S5, which was performed using MATLAB.

Estimation of reporter-positive cell burden per CEST-voxel equivalent. For each of five reporter-transduced recipients, an exploratory estimate of the mScarlet-positive cell burden per femur was obtained by multiplying the total number of bone-marrow cells per femur by the confocal-derived mScarlet-positive fraction used as a proxy for the fraction of reporter-positive bone-marrow cells. The CEST voxel dimensions were 0.625 × 0.625 × 1 mm, corresponding to a voxel volume of 0.391 μL. The MRI-derived femoral marrow volume corresponded to approximately 38.4 CEST-voxel equivalents per femur. Estimated mScarlet-positive cells per CEST-voxel equivalent were calculated by dividing the estimated number of mScarlet-positive cells per femur by 38.4. The estimated burden ranged from 4.66×10^4 to 1.61×10^5 cells per CEST-voxel equivalent. Femoral CEST contrast was summarized as the MTRasym area under the curve integrated over 1–2 ppm. The association between estimated reporter-positive burden

and CEST contrast was evaluated using Spearman’s rank correlation coefficient and was interpreted as exploratory because only five reporter-transduced recipients were included.

Supplementary Figures

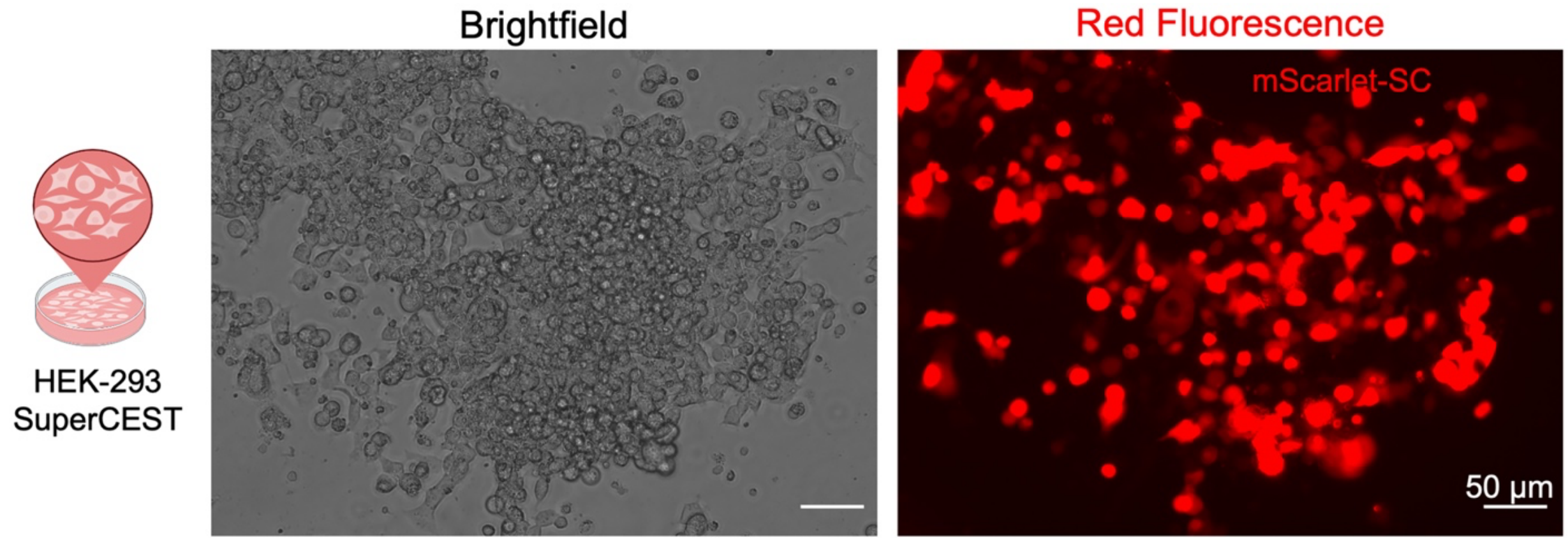

Figure S1. Representative brightfield and fluorescence images show reporter expression in HEK293T producer cells during lentiviral production. HEK293T packaging cells were transfected for lentiviral production of the dual-reporter mScarlet-SC construct. Representative brightfield and fluorescence images show robust reporter-associated red fluorescence, confirming successful expression during viral particle production.

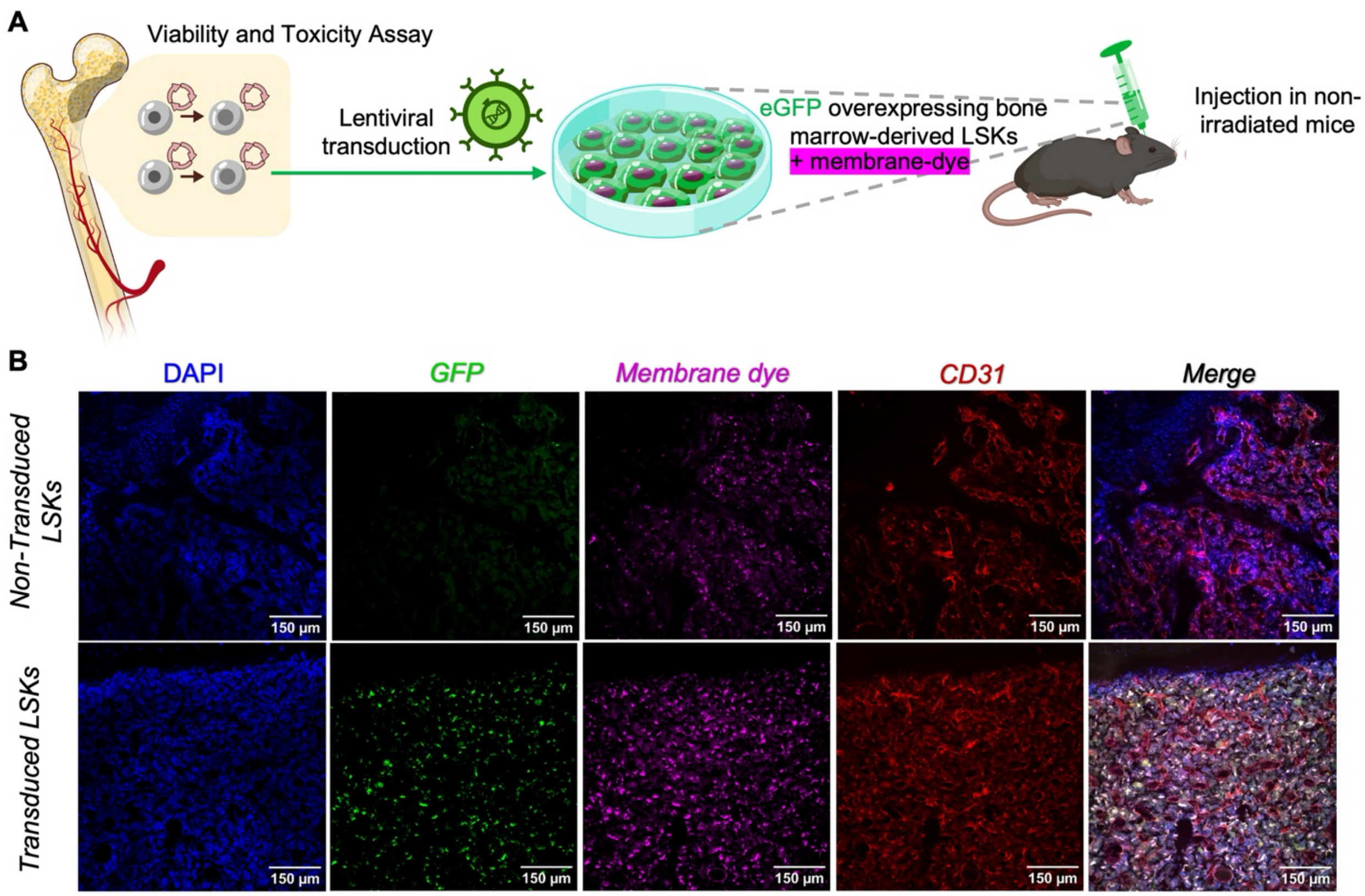

Figure S2. Short-term bone-marrow homing of genetically labeled LSK cells. (A) Experimental workflow. LSK cells were lentivirally transduced with an eGFP-expressing construct or left non-transduced, labeled with a membrane dye, and transplanted at a dose of 2.5 × 10^4 cells per mouse to assess short-term homing. **(B)** Representative femoral bone-marrow images obtained 7 days after transplantation, showing membrane-dye-positive (Membrane dye$^+$) cells in recipients of eGFP-transduced and non-transduced LSK cells. CD31-FITC delineated the bone-marrow vasculature. Membrane-dye-positive cells remained detectable in the femoral marrow 7 days after transplantation, demonstrating short-term marrow localization after ex vivo manipulation. This experiment was separate from the 8-week mScarlet-SC transplantation and CEST-MRI study.

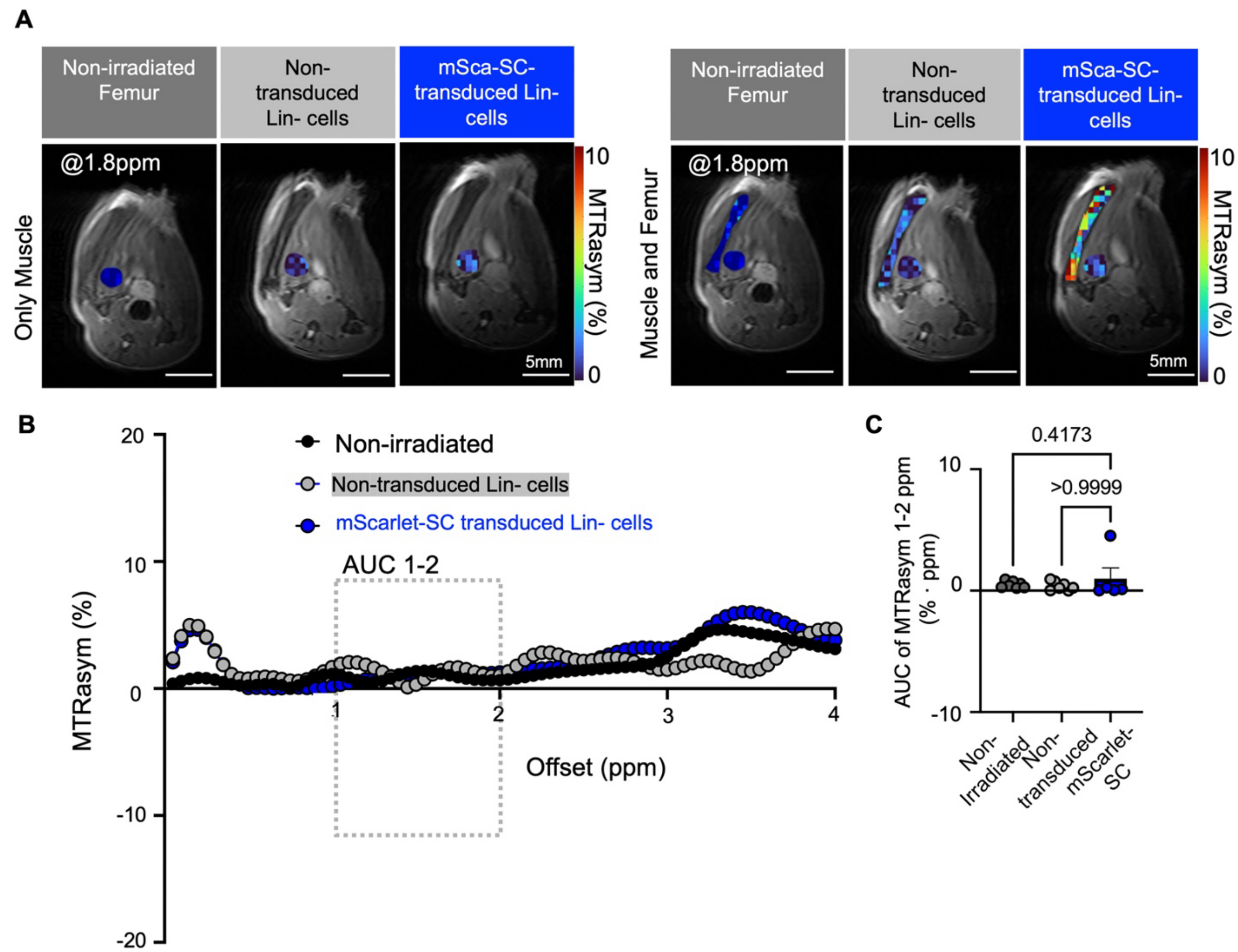

Figure S3. CEST-MRI analysis of adjacent medial thigh muscle as a within-scan spatial control. (A) Representative T2-weighted anatomical images showing the medial thigh adductor muscle ROI adjacent to the femur, with corresponding MTRasym CEST-MRI maps of the muscle alone and the muscle–femur region. **(B)** Mean ± SEM MTRasym spectra from muscle ROIs in non-irradiated controls (n=8), irradiated recipients transplanted with non-reporter-labeled Lin⁻ cells (n=7), and irradiated recipients transplanted with mScarlet-SC reporter-labeled Lin⁻ cells (n=5). **(C)** Integrated MTRasym area under the curve (AUC) from 1-2 ppm (%·ppm) for the three groups. No significant differences were detected among groups. Statistical analysis was performed using the Kruskal–Wallis test followed by Dunn's multiple-comparisons test. Each dot represents one mouse; $P < 0.05$ was considered statistically significant.

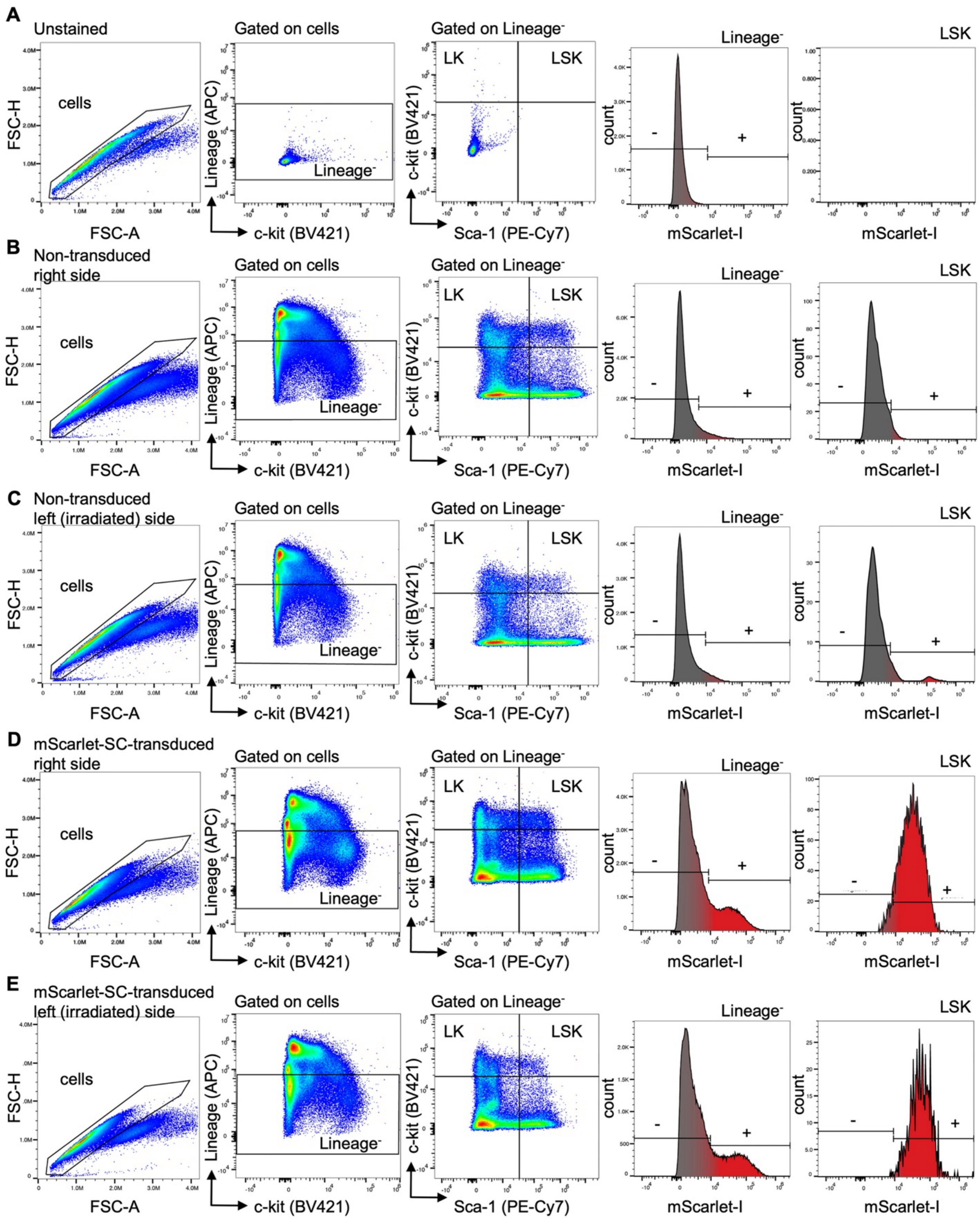

Figure S4. Flow cytometry gating strategy for ex vivo detection of reporter expression in primary tibial bone-marrow-derived hematopoietic stem and progenitor cells (HSPCs). Red fluorescence was used to identify reporter-expressing cells in samples from **(A)** an unstained

control, **(B,C)** non-transduced cells from the right and left tibiae, respectively, and **(D,E)** mScarlet-SC-transduced cells from the right and left tibiae, respectively.

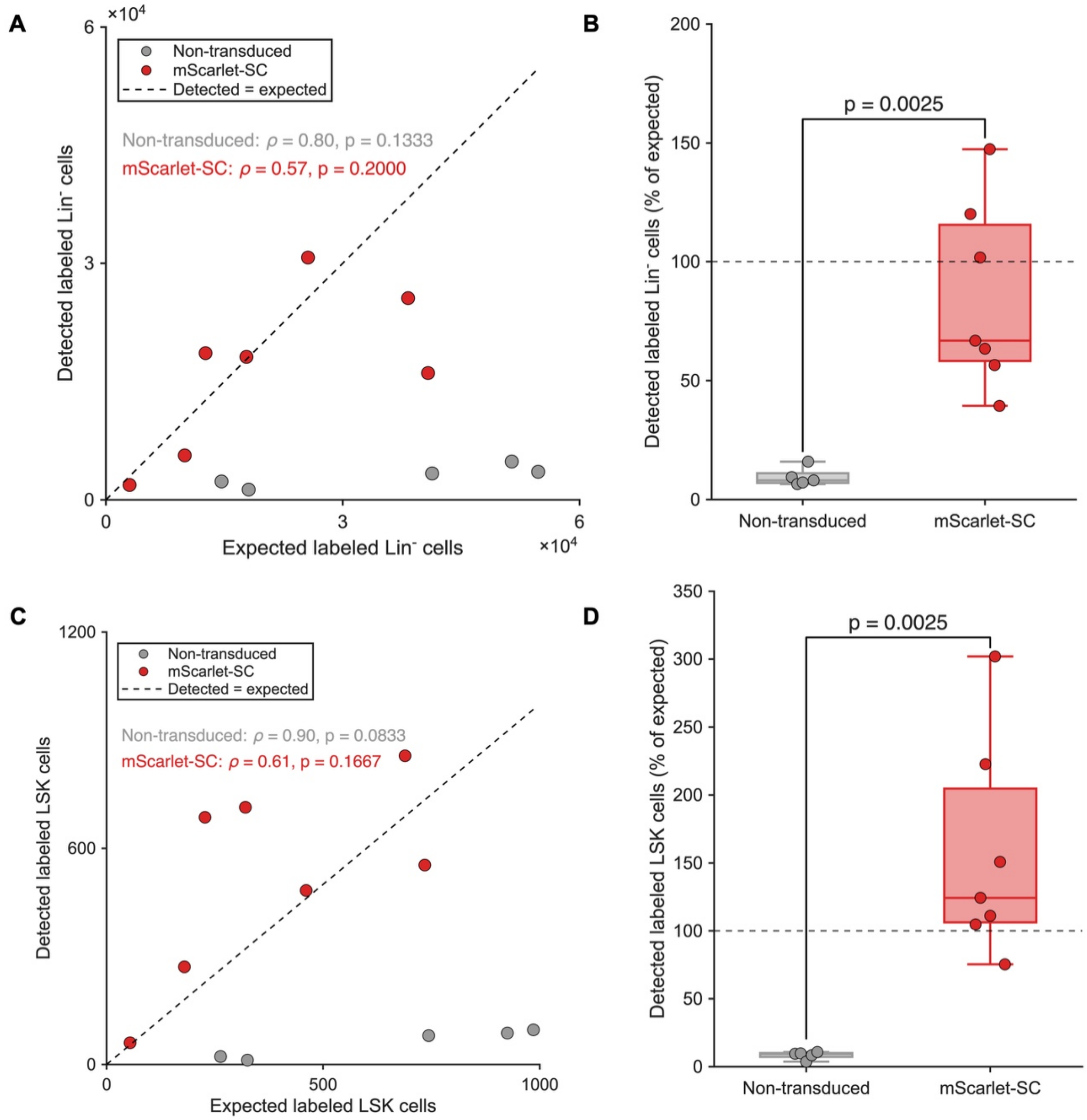

Figure S5. Comparison of detected versus expected reporter-positive Lin⁻ and LSK cells in ex vivo flow cytometry analysis of tibial bone marrow following irradiation and transplantation. (A) Relationship between detected and expected reporter-positive Lin⁻ cell numbers in non-transduced controls (gray) and mScarlet-SC-transduced samples (red). Associations were assessed separately for each group using Spearman's rank correlation coefficient (ρ) with exact two-sided permutation-based p values. The dashed identity line (y = x) indicates correspondence between expected and detected cell numbers. **(B)** Detected Lin⁻ cells expressed as a percentage of the corresponding expected values. Relative detection was higher in mScarlet-SC-transduced than in non-transduced samples (Mann-Whitney, p = 0.0025). **(C)** Relationship between expected and detected reporter-positive LSK cell numbers, analyzed as in (A). **(D)** Detected LSK cells expressed as a percentage of the corresponding expected values. Relative detection was higher in mScarlet-SC-transduced than in non-transduced samples (exact

Mann-Whitney, p = 0.0025). Each point represents a left tibial bone marrow sample from an individual mouse. In (B) and (D), boxes indicate the median and interquartile range, and the dashed horizontal line at 100% denotes equivalence between detected and expected cell numbers.

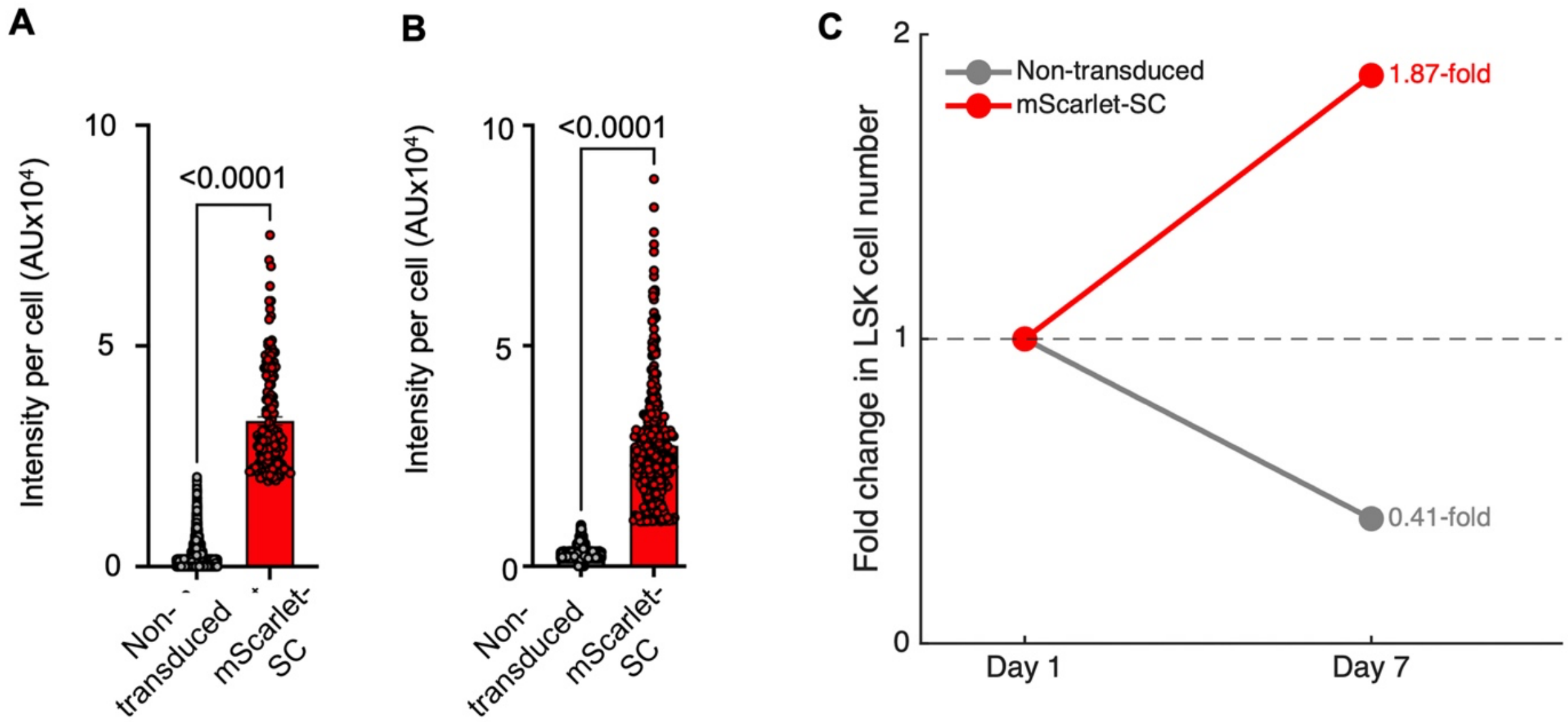

Figure S6. ImageStream analysis of reporter expression and changes in LSK cell number following transduction. Individual fluorescence intensity values of negative-control (NC) and mScarlet-SC reporter-positive LSK cells are shown at **(A)** day 1 and **(B)** day 7 following transduction. Each point represents an individual detected LSK event, and horizontal lines indicate the median. **(C)** Longitudinal change in detected LSK cell number between day 1 and day 7. Cell numbers were normalized independently within each group to the corresponding day-1 value. Negative-control LSKs decreased to 0.41-fold of the day-1 value, whereas reporter-positive LSKs increased to 1.87-fold over the same period. The longitudinal analysis is descriptive and supports maintenance of reporter-positive LSKs during the observation period.

Table S1. Exploratory estimation of reporter-positive cell burden associated with femoral CEST-MRI contrast. Per-animal estimates are shown for five mScarlet-SC-transduced recipients. The estimated number of mScarlet-positive cells per femur was calculated by multiplying total bone-marrow cellularity per femur by the confocal-derived mScarlet-positive fraction used as a proxy for the reporter-positive fraction of bone marrow. The CEST voxel dimensions were 0.625 × 0.625 × 1 mm, corresponding to a voxel volume of 0.391 µL. The MRI-derived femoral marrow volume corresponded to approximately 38.4 CEST-voxel equivalents per femur. Estimated mScarlet-positive cells per CEST-voxel equivalent were calculated by dividing the estimated number of mScarlet-positive cells per femur by 38.4. Femoral CEST contrast is reported as the MTRasym area under the curve integrated over 1-2 ppm. These values provide an exploratory order-of-magnitude estimate and do not represent a formal detection threshold or a direct count of cells physically located within individual MRI voxels.

**Mouse**	**Total BM cells/femur**	**mScarlet$^+$ (% of total BM)**	**Estimated mScarlet$^+$ cells/femur**	**Estimated cells per marrow-equivalent CEST voxel**	**Femoral MTRasym AUC (1–2 ppm)**
1	13.0×10^6	15.3%	1.99×10^6	5.18×10^4	0.0251
2	12.6×10^6	14.2%	1.79×10^6	4.66×10^4	0.0404
3	17.0×10^6	36.4%	6.19×10^6	1.61×10^5	0.0263
4	16.0×10^6	18.9%	3.02×10^6	7.88×10^4	0.0107
5	14.0×10^6	25.8%	3.61×10^6	9.41×10^4	0.0622

Supplementary Table S2. Cross-modal correlation analysis. *P < 0.05, **P < 0.01, ***P < 0.001. Correlations were calculated using pairwise complete observations. Because of the small sample size, correlations were designated significant only when P < 0.05 and the 95% bootstrap confidence interval (CI) excluded zero. Comparisons with confidence intervals spanning zero are reported as nonsignificant (ns).

**Comparison**	**n**	**Spearman ρ**	**95% bootstrap CI**	**P value**	**Sig.**
Lin^- flow vs LSK flow	12	0.85	0.49 to 0.98	0.0005	***
Lin^- flow vs femoral CEST	12	0.74	0.32 to 0.92	0.0058	**
Lin^- flow vs in vivo IVIS	12	0.49	−0.16 to 0.88	0.1063	ns
Lin^- flow vs confocal	12	0.76	0.33 to 0.96	0.0040	**
Lin^- flow vs ex vivo IVIS	12	0.64	−0.05 to 0.93	0.0258	ns
LSK flow vs femoral CEST	12	0.85	0.51 to 0.98	0.0004	***
LSK flow vs in vivo IVIS	12	0.66	0.09 to 0.95	0.0202	*
LSK flow vs confocal	12	0.78	0.37 to 0.94	0.0030	**
LSK flow vs ex vivo IVIS	12	0.64	−0.06 to 0.93	0.0258	ns
Femoral CEST vs in vivo IVIS	12	0.80	0.32 to 0.96	0.0019	**
Femoral CEST vs confocal	12	0.92	0.67 to 0.99	0.00003	***
Femoral CEST vs ex vivo IVIS	12	0.58	−0.03 to 0.86	0.0457	ns
In vivo IVIS vs confocal	12	0.59	−0.01 to 0.91	0.0415	ns
In vivo IVIS vs ex vivo IVIS	12	0.34	−0.21 to 0.76	0.2799	ns
Confocal vs ex vivo IVIS	12	0.59	−0.04 to 0.84	0.0426	ns
